# Draft Genome of the Mirrorwing Flyingfish (*Hirundichthys speculiger*)

**DOI:** 10.3389/fgene.2021.695700

**Published:** 2021-07-07

**Authors:** Pengwei Xu, Chenxi Zhao, Xinxin You, Fan Yang, Jieming Chen, Zhiqiang Ruan, Ruobo Gu, Junmin Xu, Chao Bian, Qiong Shi

**Affiliations:** ^1^College of Life Sciences, University of Chinese Academy of Sciences, Beijing, China; ^2^Shenzhen Key Lab of Marine Genomics, Guangdong Provincial Key Lab of Molecular Breeding in Marine Economic Animals, BGI Academy of Marine Sciences, BGI Marine, BGI, Shenzhen, China; ^3^Marine Geological Department, Marine Geological Survey Institute of Hainan Province, Haikou, China

**Keywords:** flying fish, whole genome sequencing, genome assembly, *eevs*, vision-related gene, phylogenetic tree

## Summary

Flying fishes are a group of Exocoetidae members with an intriguing epipelagic inhabitant. They have evolved numerous interesting characteristics. Here, we performed whole genome sequencing, *de novo* assembly and annotation of the representative mirrorwing flyingfish (*Hirundichthys speculiger*). We obtained a 1.04-Gb genome assembly using a hybrid approach from 99.21-Gb Illumina and 29.98-Gb PacBio sequencing reads. Its contig N50 and scaffold N50 values reached 992.83 and 1,152.47 kb, respectively. The assembled genome was predicted to possess 23,611 protein-coding genes, of which 23,492 (99.5%) were functionally annotated with public databases. A total of 42.02% genome sequences consisted of repeat elements, among them DNA transposons accounted for the largest proportion (24.38%). A BUSCO (Benchmarking Universal Single Copy Orthologs) evaluation demonstrated that the genome and gene completeness were 94.2% and 95.7%, respectively. Our phylogeny tree revealed that the mirrorwing flyingfish was close to *Oryzias* species with a divergence time of about 85.2 million years ago. Moreover, nine vison-related genes, three melatonin biosynthesis related aanat (aralkylamine *N*-acetyltransferase) genes, and two sunscreen biosynthesis related *eevs* (2-epi-5-epi-valiolone synthase) genes were identified in the assembled genome; however, the loss of *SWS1* (short-wavelength sensitive opsin 1) and aanat1a in amphibious mudskippers was not presented in the mirrorwing flyingfish genome. In summary, we generate a high-quality draft genome assembly for the mirrorwing flyingfish, which provides new insights into physiology-related genes of Exocoetidae. It also serves as a powerful resource for exploring intriguing traits of Exocoetidae at a genomics level.

## Introduction

Flying fishes (Exocoetidae; Beloniformes) have evolved with numerous interesting characteristics, such as gliding over water, marine- to freshwater transition, and unique craniofacial and egg buoyancy. They have been regarded as an extraordinary marine group with enlarged pelvic fins and hypocercal caudal fins, which could help to glide over water to reach a distance up to 400 m (Davenport, 1994). Although the oldest gliding fish fossil (*Potanichthys xingyiensis*) shares certain similar morphology with modern flying fishes, it is not the ancestor of the modern flying fishes, since they are thought to have evolved independently about 65.5 million years ago (Xu et al., 2012). Compared with tetrapod gliders, the gliding behavior of flying fishes could not be considered as an energy-saving strategy for long-distance movement (Rayner, 1986), but it may be just used for escaping from underwater predators [e.g., swordfish, tuna, dolphin, and squid (Kutschera, 2005)].

**Figure 1:**
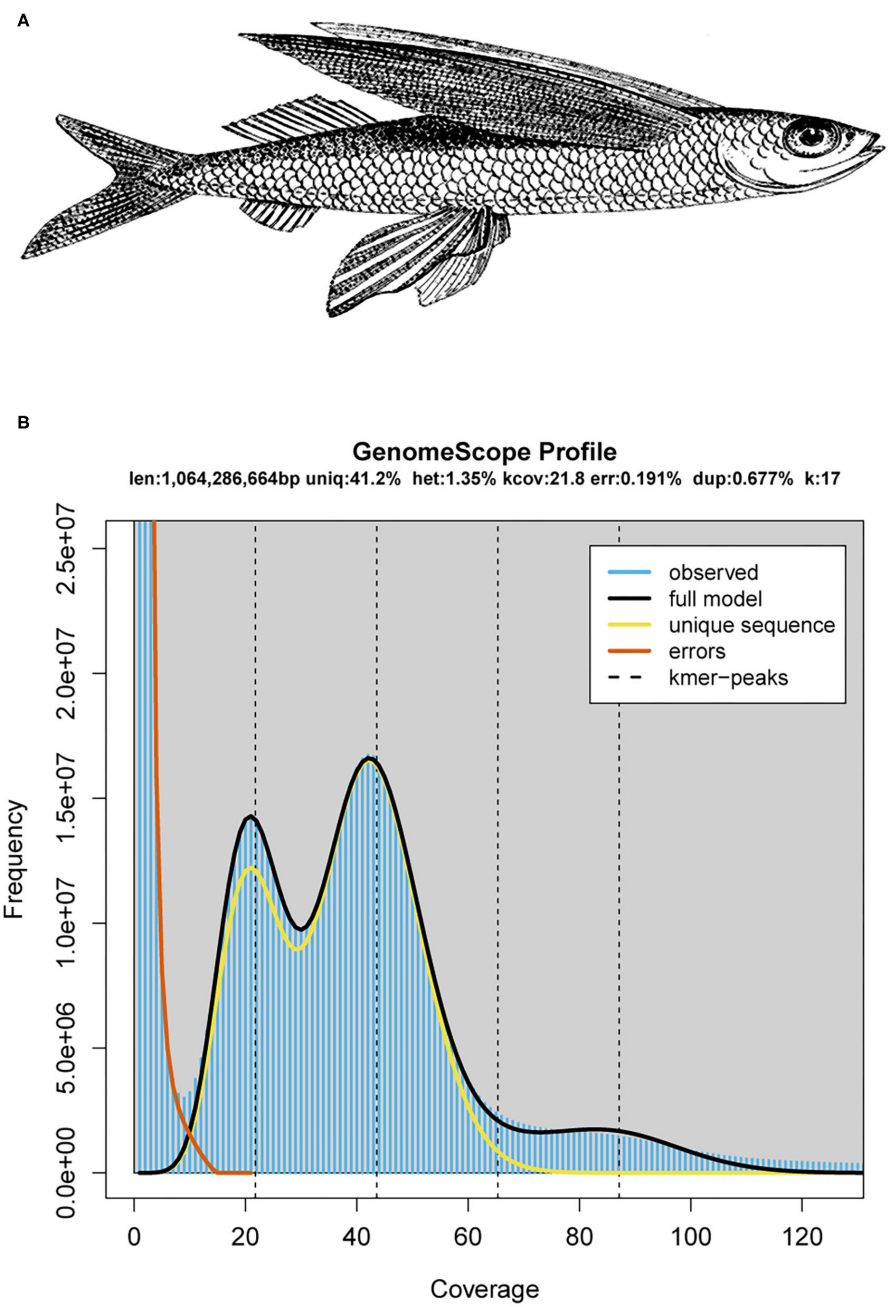
The schematic diagram and genomics feature of the mirrorwing flyingfish. **(A)** A drawing of the mirrorwing flyingfish [adopted from De Bruin et al. (1995)]. **(B)** A *k*-mer analysis of the genome sequencing reads for the mirrorwing flyingfish using GenomeScope.

While the representative mirrorwing flyingfish (*Hirundichthys speculiger*; Figure 1A) traverses the air and water interface, it meets a series of challenges [such as relentless sunshine, lack of buoyancy, and high CO_2_ accumulation (Wright and Turko, 2016)] as amphibious fishes. The lower refractive index of air usually aggravates this situation, making fishes myopic in air (Baylor and Shaw, 1962). Duplication, loss, differential expression, and crucial tuning of opsin genes could lead to visual plasticity in vertebrates for adapting to the water-to-air environments (Hauser and Chang, 2017). Five types of opsins, including LWS (red: long wavelength-sensitive), SWS1 (UV: short wavelength-sensitive 1), SWS2 (violet/blue: short wavelength-sensitive 2), RH1 (dim vision: rhodopsin), and RH2 (green: green-sensitive), have been identified in non-mammalian vertebrates (Yokoyama, 2000). Modifications of opsin and melatonin biosynthesis-related arylalkylamine *N*-acetyltransferase (*aanat*) genes could enhance amphibious mudskippers' survival on land (You et al., 2014). When the mirrorwing flyingfish leaps out of water, whether it employs the same mechanisms as mudskippers (including crucial mutation sites of LWS, lack of SWS1, and loss of *aanat1a* in the giant-fin mudskipper; see more details in You et al., 2014) or not is still an open question.

Ultraviolet radiation (UVR: 280–400 nm) often causes DNA damages through oxidative stress, producing a number of disorders (such as sunburn and skin cancer risk) (Kageyama and Waditee-Sirisattha, 2019; Rosic, 2019). UV-absorbing compounds, such as mycosporine-like amino acids (MAAs) and gadusol, are commonly distributed in various marine microorganisms, invertebrates, and algae (Shick and Dunlap, 2002; Miyamoto et al., 2014). The *de novo* synthesis of MAA in invertebrates (such as coral and sea anemone) employed a four-step desmethyl-4-deoxygadusol synthase (DDGS) based pathway as cyanobacteria (Balskus and Walsh, 2010; Rosic and Dove, 2011; Shinzato et al., 2011), while zebrafish (*Danio rerio*) could convert sedoheptulose-7-phosphate (SH7P) to gadusol using 2-epi-5-epi-valiolone synthase (EEVS) and S-adenosyl-L-methionine-dependent methyltransferase [MT-Ox (Osborn et al., 2015)]. The two core genes, *eevs* and *mt-ox*, in zebrafish are flanked by four transcription factor genes [*frmd4B, mitf* , *mdfic*, and *foxp1* (Osborn et al., 2015)], which is not consistent with the loss of *mdfic* in Japanese medaka [*Oryzias latipes* (Kim et al., 2018)]. Phylogenetic analysis using mitochondrial genes in Beloniformes had inferred a close relationship between the mirrorwing flyingfish and medaka (Lovejoy et al., 2004; Cui et al., 2018). Whether the mirrorwing flyingfish contains the complete gene cluster as zebrafish or incomplete cluster as medaka is valuable for checking the possible lineage-specific gene rearrangement of *eevs*-like cluster.

Here, we performed whole genome sequencing of the mirrorwing flyingfish and generated a draft assembly with a hybrid method (Ye et al., 2016) for the first time. Our subsequent phylogenetic and comparative genomic analyses between amphibious fishes and ordinary underwater fishes will provide insights into the evolution of vision-related genes, olfactory receptor (OR) genes, and gadusol synthesis-related genes (*eevs*) in the mirrorwing flyingfish. This genome assembly will serve as a valuable resource for the illumination of molecular basis for the special characteristics of flying fishes.

### Value of the Data

This is the first genome report of the representative mirrorwing flyingfish. Our final assembly was 1.04 Gb, with a contig N50 of 992.83 kb and a scaffold N50 of 1,152.47 kb.

A phylogeny tree was constructed to demonstrate that the mirrorwing flyingfish was close to *Oryzias* species with a divergence time of about 85.2 Mya. A total of 60.71% of the mirrorwing flyingfish genome region was syntenic with *O. latipes*.

The genome of mirrorwing flyingfish harbored nine vision-related genes, three *aanat* genes, and two *eevs*-like genes. The existence of *SWS1* and *aanat1a* suggests that the mirrorwing flyingfish employs different strategies for visional adaptation in air. A gene cluster of *eevs*-like shared the same synteny as Japanese medaka, implying a uniform gene rearrangement in Beloniformes.

## Materials and Methods

### Fish Sampling and Genome Sequencing

An adult mirrorwing flyingfish was captured by torch fishing in the water area of Iltis Bank, Xisha, China. Genomic DNAs were extracted from muscle tissues and purified and quality checked according to a standard protocol (Sigma-Aldrich, St. Louis, MO, USA).

Subsequently, three paired-end libraries (with insert sizes of 270, 500, and 800 bp, respectively) and three mate-pair libraries (with insert sizes of 2, 5, and 10 kb, respectively) were constructed in accordance with an Illumina standard manual before sequencing on an Illumina X-Ten platform (Illumina Inc., San Diego, CA, USA) with a PE-150 or PE-125 module. Raw reads were then processed using SOAPnuke v1.5.6 (Chen et al., 2018) with optimized parameters (“-n 0.02 -Q 2 -l 15−5 1 -d -I -q 0.4”). An additional SMART Bell library with an insert size of 20 kb was constructed based on a PacBio RS II protocol (Pacific Biosciences, Menlo Park, CA, USA). Six DNA sequencing cells were produced using the P6 polymerase/C4 chemistry (Rhoads and Au, 2015).

### Genome Assembly

Distribution of *k-mer* frequency was constructed with jellyfish v2.0 (Marçais and Kingsford, 2011) using clean reads from short-insert libraries (270 and 500 bp). GenomeScope v1.0 (Vurture et al., 2017) was then applied to estimate the genome size and heterozygosity. A routine hybrid pipeline was employed to assemble the high heterozygous flyingfish genome (Supplementary Figure 1).

In brief, the Illumina paired-end reads were first assembled using Platanus v1.24 (Kajitani et al., 2014) with optimized parameters (assemble -k 35 -s 5 -u 0.2 -d 0.5). DBG2OLC (Ye et al., 2016) was employed to construct backbone sequences from the best overlaps between the initial contigs and raw PacBio reads. All related PacBio reads were realigned to the backbone with Sparc (Ye and Ma, 2016) to construct the most likely consensus sequences of the genome. All Illumina paired-end reads were aligned to the resulting assembly using BWA-MEM (Li, 2014). The alignments were employed for Pilon v1.24 (Walker et al., 2014) to polish the assembly. All Illumina mate-pair reads were mapped onto the corrected contigs using BWA-MEM (Li, 2014). These alignments were then processed with BESST v2.2.4 (Sahlin et al., 2014) to construct scaffolds. Completeness of the genome assembly was evaluated by BUSCO v3.0 (Simão et al., 2015) with default parameters “-l actinopterygii_odb9 -m genome -c 3 -sp zebrafish.”

### Genome Annotation

Transposable elements (TEs) were identified using both homolog-based and *de novo* methods. For the homolog-based method, RepeatMasker v4.06 and ProteinRepeatMasker v4.06 (Chen, 2004) were employed to identify known TEs against the Repbase v21.0 (Jurka et al., 2005). For the *de novo* method, a *de novo* library was constructed using RepeatModeler v2.0 (Flynn et al., 2020) and LTR-FINDER v1.0.6 (Xu and Wang, 2007) firstly. Then, RepeatMasker v4.06 was subjected to identify the *de novo* TEs against the *de novo* library. The tandem repeat sequences were identified using Tandem Repeat Finder (Benson, 1999).

Gene models were also predicted using both homolog-based and *de novo* methods. For the homolog-based methods, protein sequences of zebrafish (*Danio rerio*), three-spined stickleback (*Gasterosteus aculeatus*), human (*Homo sapiens*), Japanese medaka (*O. latipes*), and green spotted pufferfish (*Tetraodon nigroviridis*) were derived from Ensembl-100 and aligned to our flyingfish genome using tBLASTn (Ye et al., 2006) with parameter “-e 1e-5 -m 8 -F.” Blasted hits were processed by SOLAR v0.9 (Yu et al., 2006) with parameter “-a prot2 genome2 -z” to determine the potential gene loci. We extracted the candidate gene region with 2-kb flanking sequences and employed Genewise v2.4 (Birney et al., 2004) to determine gene structures. For the *de novo* prediction, we trained the parameters of AUGUSTUS v3.2 (Stanke et al., 2006) using randomly selected 2,000 intact gene models that were derived from the homolog-based method. Then, we used AUGUSTUS to perform *ab initio* prediction on the repeat-masked genome with the trained parameters. Finally, the gene models predicted from both approaches were integrated to form non-redundant gene sets using the similar pipeline as described in a previous study (Xiong et al., 2016). Completeness of the gene sets was evaluated by BUSCO v3.0 (Simão et al., 2015) with parameters “-l actinopterygii_odb9 -m protein -c 3 -sp zebrafish.”

Gene function annotation was performed on the basis of sequence and domain similarity. The protein sequences were aligned to Kyoto Encyclopedia of Genes and Genomes (KEGG) v84.0 (Kanehisa et al., 2017), SwissProt, and TrEMBL (Uniprot release 2020-06) (Bairoch et al., 2005) using BLASTP (Ye et al., 2006) with an E-value of 1e−5. InterProScan v5.11-55.0 (Jones et al., 2014) was applied to predict domain information with public databases including Pfam (Bateman et al., 2004), SMART (Letunic et al., 2012), PANTHER (Thomas et al., 2003), PRINTS (Attwood et al., 2000), PROSITE profiles (Sigrist et al., 2010), and ProDom (Servant et al., 2002). Gene Ontology (GO) terms were predicted using the IPR entry list (Burge et al., 2012).

Four types of non-coding RNA were identified in the mirrorwing flyingfish genome. We employed tRNAscan-SE v2.0 (Lowe and Eddy, 1997) to detect transfer RNAs (tRNAs). For microRNAs (miRNAs) and small nuclear RNAs (snRNAs), the Rfam v12.0 (Nawrocki et al., 2015) database was mapped onto the assembled genome, and the matched sequences were delivered into INFERNAL v1.1.4 (Nawrocki and Eddy, 2013) to confirm structures. Ribosomal RNAs (rRNAs) in the genome were searched using animal full-length rRNAs (Quast et al., 2012) as the query.

### Gene Family Prediction

To identify gene families in the mirrorwing flyingfish genome, we download protein-coding sequences of 18 representative teleost fishes from the National Center for Biotechnology Information (NCBI) databases (see more details in Supplementary Table 1), including *Anabas testudineus* (Ates; climbing perch), *Austrofundulus limnaeus* (annual killifish), *Boleophthalmus pectinirostris* (Bpec; great blue-spotted mudskipper), *Channa argus* (Carg; northern snakehead), *Cyprinodon variegatus* (sheepshead minnow), *D. rerio* (Drer; zebrafish), *Fundulus heteroclitus* (mummichog), *Kryptolebias marmoratus* (Kmar; mangrove rivulus fish), *Monopterus albus* (Asian swamp eel), *Nothobranchius furzeri* (turquoise killifish), *Oreochromis aureus* (Oaur; blue tilapia), *O. niloticus* (Onil; Nile tilapia), *Oryzias latipes* (Olat; Japanese medaka), *O. melastigma* (Omel; marine medaka), *Periophthalmus magnuspinnatus* (Pmag; giant-fin mudskipper), *Poecilia mexicana* (Atlantic molly), *Xiphophorus maculatus* (southern platyfish), and *Maylandia zebra* (Mzeb; *Zebra mbuna*). After removal of alternative splice variants, the protein sequences of the 18 fish species along with the mirrorwing flyingfish (*H. speculiger*; Hspe) were delivered to OrthoFinder v2.3.11 (Emms and Kelly, 2019) with an E-value of 1e−5 to identify orthologous groups.

Protein sequences of single-copy orthologous families were extracted and aligned using MUSCLE v3.8 (Edgar, 2004), and the alignment of protein sequences was converted to codon alignment using PAL2NAL v14 (Suyama et al., 2006). The phase 1 sites of codon aligned were extracted and concentrated to a super gene for each species. PhyML v3.0 (Guindon et al., 2010) and MrBayes v3.2 (Ronquist et al., 2012) were employed to construct a phylogenetic tree. Divergence time of these teleost fishes was estimated using MCMCTREE v4.5 in the PAML v4.5 (Yang, 2007) with five putative calibrations times, which were adapted from TIMETREE (Kumar et al., 2017). We used CAFÉ v3.0 (Han et al., 2013) with optimized parameter (-p 0.05 -t 4 -r 10000 -filter) to assess expansion and contraction of gene families. A branch specific *p* < 0.05 was utilized to define significance in the mirrorwing flyingfish. We employed hypergeometric tests (Falcon and Gentleman, 2008) to investigate pathway enrichments of those significantly expanded gene families, using the whole genome annotation as the background.

### Synteny Analysis With Medaka and Zebrafish Genomes

After masking transposon elements of the three genomes, pairwise genome alignment among mirrorwing flyingfish, Japanese medaka, and zebrafish was carried out using LASZT v1.04.03 (Harris, 2007) with optimized parameters (*T* = 2 *C* = 2 *H* = 2000 *Y* = 3400 *L* = 6000 *K* = 2200 –format = axt). The matching length of each pairwise alignment was calculated using an in-house Perl script.

### Identification of Vision-Related Genes

We applied two approaches to obtain the protein sequences of various opsins and *aanat* genes in 12 representative teleost fishes (with abbreviations of Ates, Bpec, Carg, Drer, Kmar, Mzeb, Oaur, Onil, Olat, Omel, Pmag, and Hspe, respectively, in Supplementary Table 1). For those with public annotations, gene sequences were directly downloaded from NCBI (Supplementary Table 2). For the mirrorwing flyingfish, however, we mapped the protein sequences of blue tilapia, zebrafish, and Japanese medaka to our assembled genome and predicted opsin and *aanat* genes using Exonerate v2.2.0 (Slater and Birney, 2005) with optimized parameters (-model protein2genome –showalignment false –showtargetgff true –bestn 1).

To validate the synteny of opsin genes, we downloaded those genes that have been reported to locate adjacent to an opsin gene (Lin et al., 2017) and obtained the neighboring genes from the genome annotation or using BLAST with an E-value of 1e−5 against the assembled genome. We constructed a rooted neighbor-joining (NJ) tree of opsins, using known opsin from human (ENSP00000358967.4, LWS1; ENSP00000472316.1, MWS; ENSP00000358945.4, MWS2; ENSP00000469970.1, MWS3; ENSP00000296271.3, RH1; ENSP00000249389.2, SWS1) and zebrafish (ENSDARP00000069184.5, OPN3; as the outgroup) by MEGA-X (Kumar et al., 2018) with 1,000 bootstraps.

A phylogenetic tree of *aanat* gene family was also constructed using the NJ method as implemented in the MEGA-X with human AANAT (NP_001079.1) and mouse AANAT (NP_033721.1) as the outgroup (Kumar et al., 2018). We applied Evolview (Subramanian et al., 2019) to edit phylogenetic trees. Five key tuning sites (including 180, 197, 277, 285, and 308) of the LWS opsins had influenced the λ_max_ of vertebrate opsins (Bowmaker, 2008; Yokoyama, 2008). A previous report suggested that a single mutation at S180A, H197Y, Y277F, T285A, A308S, and double mutations S180A/H197Y can lead to a −7, −28, −8, −15, −27, and −11 nm shift, respectively, in the λ_max_ of the pigments (Yokoyama and Radlwimmer, 2001). To investigate classical five key tuning sites of LWS, we obtained the global alignment of LWS in 12 teleost fishes and human being using MUSCLE v3.8 (Edgar, 2004) and highlighted the five crucial sites with Jalview v2.11.1.3 (Waterhouse et al., 2009). F86 of SWS1 opsin is crucial for UV sensing; the mutation of F86V in goldfish led to +1 nm shift in the absorption spectrum of the SWS1 opsins (Tada et al., 2009). The tuning site F86 resulting in the UV perception of SWS1 opsin in vertebrates (Hunt et al., 2007) was also checked in SWS1-containing teleost fishes.

### Characterization of Gadusol Biosynthesis Genes

To identify gadusol biosynthesis related genes, we extracted the *eevs*-like and *mt-ox* genes and genes adjoined to them in zebrafish, tilapia, and medaka genomes that were collected from the NCBI database (Supplementary Table 3) as the references and employed the same method as mentioned for the vision-related genes to predict *eevs*-like and *mt-ox* in in the mirrorwing flyingfish genome. For other 11 selected teleost fishes, we retrieved *eevs*-like and *mt-ox* from the NCBI annotation. We constructed a rooted NJ tree using a dehydroquinate synthase (DHQS-like) derived from cyanobacteria (Balskus and Walsh, 2010) as the outgroup by MEGA-X with 1,000 bootstraps. Conserved domains and motifs of the candidate *eevs*-like genes were predicted using the NCBI Conserved Domain Database (CDD) (Lu et al., 2020) and MEME website server (Bailey et al., 2006), and then, TBtools suite was applied to illuminate the phylogenetic tree, conserved domains, and motifs (Chen et al., 2020).

### Identification of Olfactory Receptor Genes

Reference sequences of olfactory receptor (OR) genes were obtained from a previous paper (Niimura, 2009). The full-length OR protein sequences were aligned to nine teleost fishes (including Ates, Bpec, Pmag, Carg, Kmar, Hspe, Drer, Oaur, and Olat) using tBLASTn (Ye et al., 2006) with an E-value of 1e−5, and the blasted hits were clustered using SOLAR v0.9 (Yu et al., 2006) to define candidate gene loci.

We extracted these candidate gene loci along with 2-kb flank region and employed GeneWise v2.4 (Birney et al., 2004) to predict gene structures. First, the potential OR genes without start/stop codons or with interrupting stop codon(s) or frameshift(s) were excluded. Second, the full-length sequences were inspected using the NCBI non-redundant database (BLASTP with an E-value of 1e−5), but those candidate OR genes with the best hit annotation of non-OR were discarded. Finally, the remaining sequences were further checked using TMHMM v2.0 (Krogh et al., 2001) to identify the putative seven transmembrane domains. We aligned the protein sequences of confirmed OR genes using MUSCLE in the MEGA-X (Kumar et al., 2018) and then constructed a rooted neighbor-joining tree using human G-protein coupled receptor 35 (NP_005292.2) and human G-protein coupled receptor 132 (NP_037477.1) as the outgroup by MEGA-X with the Poisson model and uniform rates.

## Results and Discussion

### Summary of the Genome Assembly and Annotation

The Illumina sequencing generated a total of ~138.13-Gb raw reads, and then, 99.21-Gb clean reads were retained after filtering low-quality sequences (Supplementary Table 4). The PacBio sequencing yielded about 29.98-Gb data, consisting of 2,785,344 reads with an N50 length of 16.5 kb (Supplementary Table 5).

A *k-mer* analysis predicted that the mirrorwing flyingfish had an estimated genome size of 1.06 Gb and a heterozygosity of 1.35% (Figure 1B). After contig building, consensus calling, polishing, and scaffold construction, we generated a final assembly of 1.04 Gb, which is nearly equal to the estimated genome size. The draft assembly consisted of 3,052 scaffolds (> 650 bp in length), and the contig and scaffold N50 values of our final assembly were 992.83 and 1,152.47 kb (Table 1).

**Table 1:** Statistics of our genome assembly.

**Parameter**	**Platanus contig**		**DBG2OLC**		**Pilon**		**BESST**	
	**Size (bp)**	**Number**	**Size (bp)**	**Number**	**Size (bp)**	**Number**	**Size (bp)**	**Number**
N90	131	3,1,85,718	113237	1567	112663	1,567	161399	1,205
N80	161	2,2,24,282	235476	939	233652	939	318262	745
N70	212	1,4,16,513	396517	597	394432	597	513435	485
N60	315	8,49,429	635760	385	630993	385	831356	322
N50	514	4,85,451	998191	257	992826	257	1152470	215
Longest	36570	—————	6848566	————	6813063	————	9488118	—————
Total Size	1442411998	—————	1047997551	————	1042531442	————	1043046751	—————
> =100bp	—————	4,47,1742	—————	3852	—————	3,852	—————	3,052
> =2kb	—————	98,312	—————	3849	—————	3,849	—————	3,049

*Platanus: primary contig assembly using Platanus; DBG2OLC: call consensus with blasr and the consensus module (sparc) using the previous result and PacBio subreads; Pilon: polish DBG2OLC result with pair-end reads; BESST: scaffold construct with mate-pair reads*.

The BUSCO evaluation indicated that 94.2% of the Actinopterygii gene sets were identified as complete (4,317 out of 4,584, actinopterygii_odb9) in the mirrorwing flyingfish genome (Table 2). We also assessed accuracy of the draft assembly by mapping Illumina paired-end reads onto the assembled genome sequences. A total of 94.91% of the Illumina paired-end reads were properly mapped to the assembled genome, with a good coverage of 97.78% (Supplementary Table 6). The high completeness of BUSCOs and nucleotide-level accuracy, together with considerable continuity of contig sizes, suggested that our high-quality genome assembly could be qualified for further data analysis.

**Table 2:** Evaluation of the genome and gene completeness with BUSCO.

**BUSCO**	**Genome**		**Gene**	
	**Numbers**	**Percent (%)**	**Numbers**	**Percent (%)**
Total BUSCOs	4,584			
Complete BUSCOs	4,317	94.2	4,386	95.7
Complete and single-copy BUSCOs	4,074	88.9	4,103	89.5
Complete and duplicated BUSCOs	243	5.3	283	6.2
Fragmented BUSCOs	108	2.4	130	2.8
Missing BUSCOs	159	3.4	68	1.5

Repeat content of the mirrorwing flyingfish genome was calculated by combination of both homolog-based and *de novo* methods. We determined that repeat elements occupied 42.02% of the assembled genome, and DNA transposons accounted for the largest proportion (24.38%) of transposable elements (TEs; Supplementary Table 8). A total of 8.19% of the mirrorwing flyingfish genome sequences were composed of tandem repeat elements (Supplementary Table 7). Divergence rates of the TEs in the mirrorwing flyingfish genome were determined using Repbase and *de novo* libraries, respectively. We observed that 10.72 Mb of identified TEs had a <10% divergence rate from the Repbase consensus; 277.08 Mb of TE sequences (26.56% of the assembly genome) had a <10% divergence rate from the *de novo* library (Supplementary Figure 2), which were possible to be active with a recent origin.

We predicted 23,611 protein-coding genes in the mirrorwing flyingfish genome, with an average gene length of 14.35 kb. Moreover, 99.50% of these genes could be functionally annotated by at least one of the four popular databases, with 20,692 KEGG hits, 21,453 SwissProt hits, 23,477 TrEMBL hits, and 21,888 Interpro hits (Supplementary Table 9). Additionally, the BUSCO evaluation of genes demonstrated that 95.7% of the Actinopterygii gene sets were predicted as complete (4,386 out of 4,584 actinopterygii_odb9) in the mirrorwing flyingfish gene set (Table 2), suggesting high quality of our gene prediction. Furthermore, we identified four types of non-coding RNA, 247 miRNAs, 2,138 tRNAs, 538 rRNAs, and 298 snRNAs in the assembled genome (Supplementary Table 10).

### Gene Families and Phylogeny

Our gene family data demonstrated that protein-coding sequences in the 19 teleost fishes were clustered into 22,669 gene families, of which 4,632 families were 1:1 single-copy orthologs. A total of 93.5% (22,083 out of 23,611) of the mirrorwing flyingfish protein-coding genes were grouped into 17,352 gene families (Supplementary Table 11), defining 7,335 single-copy orthologs and 323 unique paralogs (Supplementary Figure 3B).

Using the 4,632 1:1 single-copy orthologous genes, we established a coincident phylogenetic topology with the ML and Bayes methods (Supplementary Figures 4, 5). The divergence tree revealed that the flyingfish was close to the two medaka species with a divergence time of about 85.2 Mya (Supplementary Figure 6). A total of 60.71% (633.32 Mb) of the mirrorwing flyingfish genome was syntenic with Japanese medaka, while only 14.66% (152.94 Mb) of the mirrorwing flyingfish genome shared synteny with zebrafish (see more details in Supplementary Table 12).

We identified 1,236 expanded gene families and 1,539 contracted gene families in the mirrorwing flyingfish genome (Supplementary Figure 3A). Among them, 135 and 131 were significantly expanded and contracted (*p* < 0.05). The KEGG enrichment analysis demonstrated that those genes belonging to the expanded gene families were related to signaling molecules and interaction, nervous system, and immune system (Supplementary Table 13, *p* < 0.01).

### Various Vision-Related Genes in the Mirrorwing Flyingfish

Vision plays a vital role in animal life, affording an important ability to perceive environmental stimuli. The visual ability of this animal depends on the numbers of opsin proteins (Bowmaker, 2008). Various fishes have accommodated a wide range of habitats (such as freshwater and marine, stagnant and running water, and shallow and deep sea), which provide differential vision adaptation (Hauser and Chang, 2017). We classified 12 teleost fishes into three groups in terms of living habitat, including genuine amphibious inhabitant (Ates, Bpec, Pmag, Carg, Kmar), normal underwater dweller (Drer, Oaur, Onil, Mzeb, Olat, Omel), and temporary water surface traveler (Hspe), for comparison of the variations among opsin proteins.

**Figure 2:**
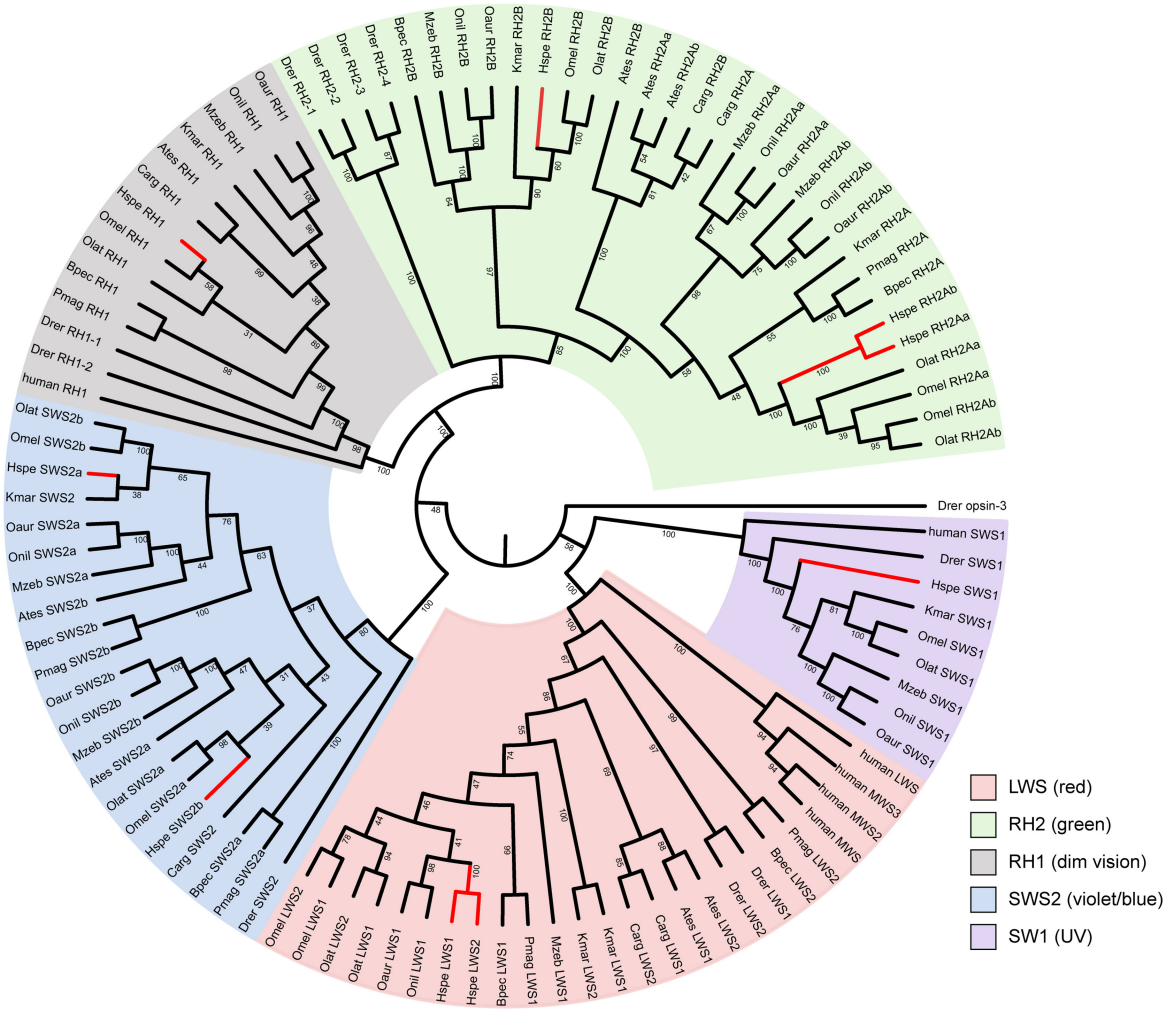
The phylogenetic tree of vertebrate opsin genes. A rooted neighbor-joining (NJ) tree was constructed with zebrafish opsin3 as the outgroup. Abbreviations are provided in Supplementary Table 1.

The mirrorwing flyingfish genome contains five types of opsins, with two LWS, two SWS2, one SWS1, one RH1, and three RH2 (Figure 2; Table 3). The maximal absorption spectra (λ_max_) of flyingfish LWS, based on the popular “five-sites” rule (You et al., 2014), are predicted to be 560 nm, which is similar to the parameters in climbing perch, northern snakehead, mangrove rivulus, blue tilapia, Nile tilapia, zebra mbuna, Japanese medaka, and marine medaka (Supplementary Table 14). The five crucial sites of LWS in the mirrorwing flying fish are 180S, 197H, 277Y, 285T, and 308A (Supplementary Figure 7).

**Table 3:** Copy number of vison-related genes in the 12 representative teleost fishes.

**Species**	**Common Name**	***LWS***	***SWS2***	***SWS1***	***RH1***	***RH2***	**Total**
*A. testudineus*	Climbing perch	2	2	0	1	3	8
*B. pectinirostris*	Blue-spotted mudskipper	2	2	0	1	2	7
*P. magnuspinnatus*	Giant-fin mudskipper	2	2	0	1	2	7
*C. argus*	Northern snakehead	2	1	0	1	2	6
*H. speculiger*	Mirrorwing flyingfish	2	2	1	1	3	9
*K. marmoratus*	Mangrove rivulus	2	1	1	1	2	7
*O. aureus*	Blue tilapia	1	2	1	1	3	8
*O. niloticus*	Nile tilapia	1	2	1	1	3	8
*M. zebra*	Zebra mbuna	1	2	1	1	3	8
*O. latipes*	Japanese medaka	2	2	1	1	3	9
*O. melastigma*	Indian medaka	2	2	1	1	3	9
*D. rerio*	Zebrafish	2	1	1	2	4	10

The synteny of opsins in 12 teleost fishes is quite conserved except *SWS1* (Supplementary Figures 8, 9). All amphibious fishes except mangrove rivulus fish have lost *SWS1* (Supplementary Figure 8B), which is used for UV vision. This *SWS1* missing could be related to the landing activity of these fishes. Since ultraviolet light can cause damages to the retina, the critical mutation of F86V could potentially alter absorption wave of SWS1 opsins toward violet light sensing so as to minimize the UV-induced damages (Cowing et al., 2002). These examined fishes in this study have V (valine) at 86 instead of F (phenylalanine; see Supplementary Figure 10), implying that these fishes could be UV sensing. Related amino acid numbering was based on the bovine rhodopsin sequence [GenBank accession no. M21606; (Palczewski et al., 2000)].

The five crucial sites of LWS in the mirrorwing flyingfish showed a narrow range of color sensing, demonstrating the same tendency as some amphibious fishes, such as climbing perch, northern snakehead, and mangrove rivulus fish. When these fishes move out of water, they can keep the same long-wave sensing as that in water. The *SWS1* loss events in the five examined amphibious fishes in our present study may have developed for the water-to-terrestrial adaptation; however, the reservation of *SWS1* in the mirrorwing flyingfish might be due to the short period of gliding in air instead of a real amphibious life (Davenport, 1994).

**Figure 3:**
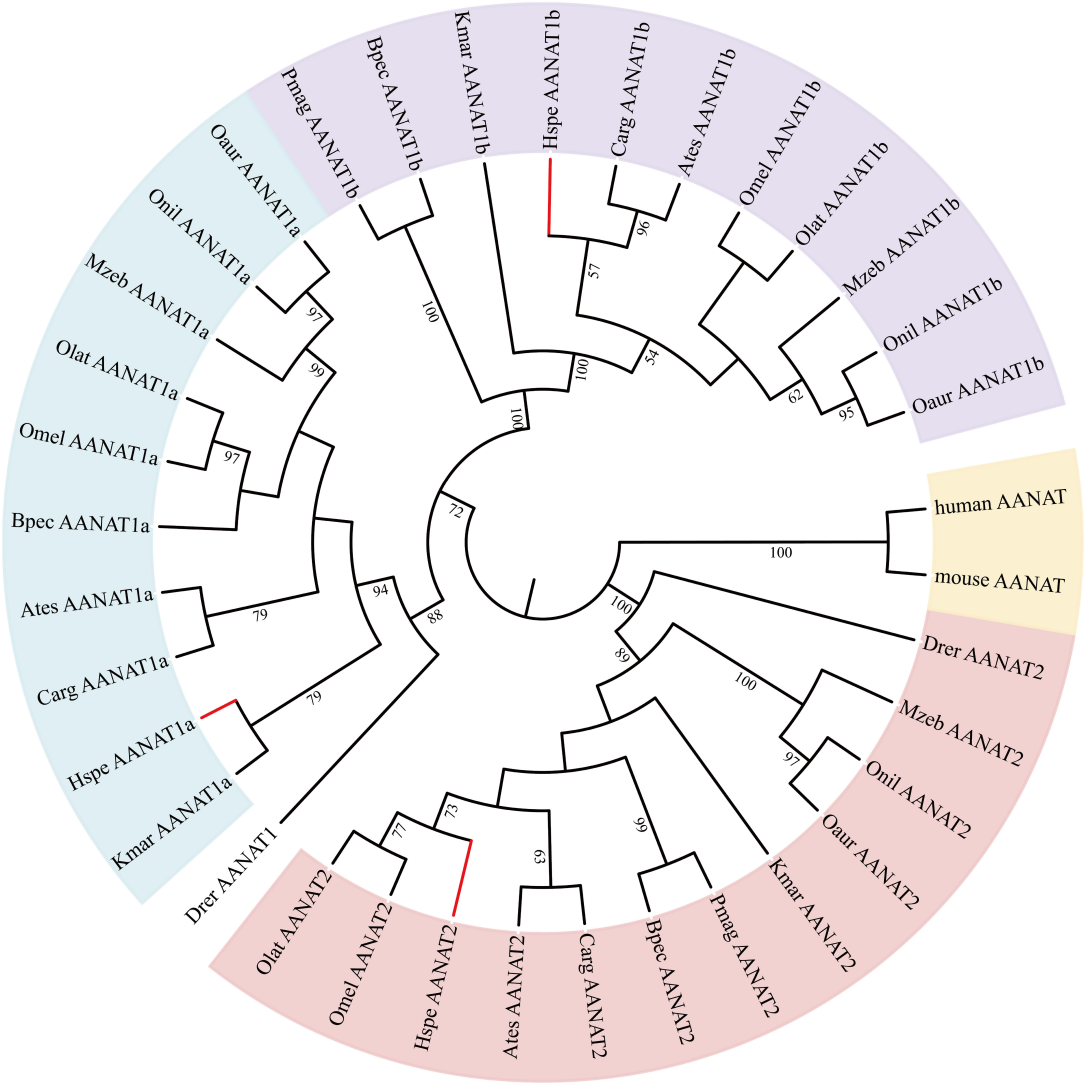
The rooted NJ tree of vertebrate *aanat* genes. It was constructed with human AANAT (NP_001079.1) and mouse AANAT (NP_033721.1) as the outgroup.

**Figure 4:**
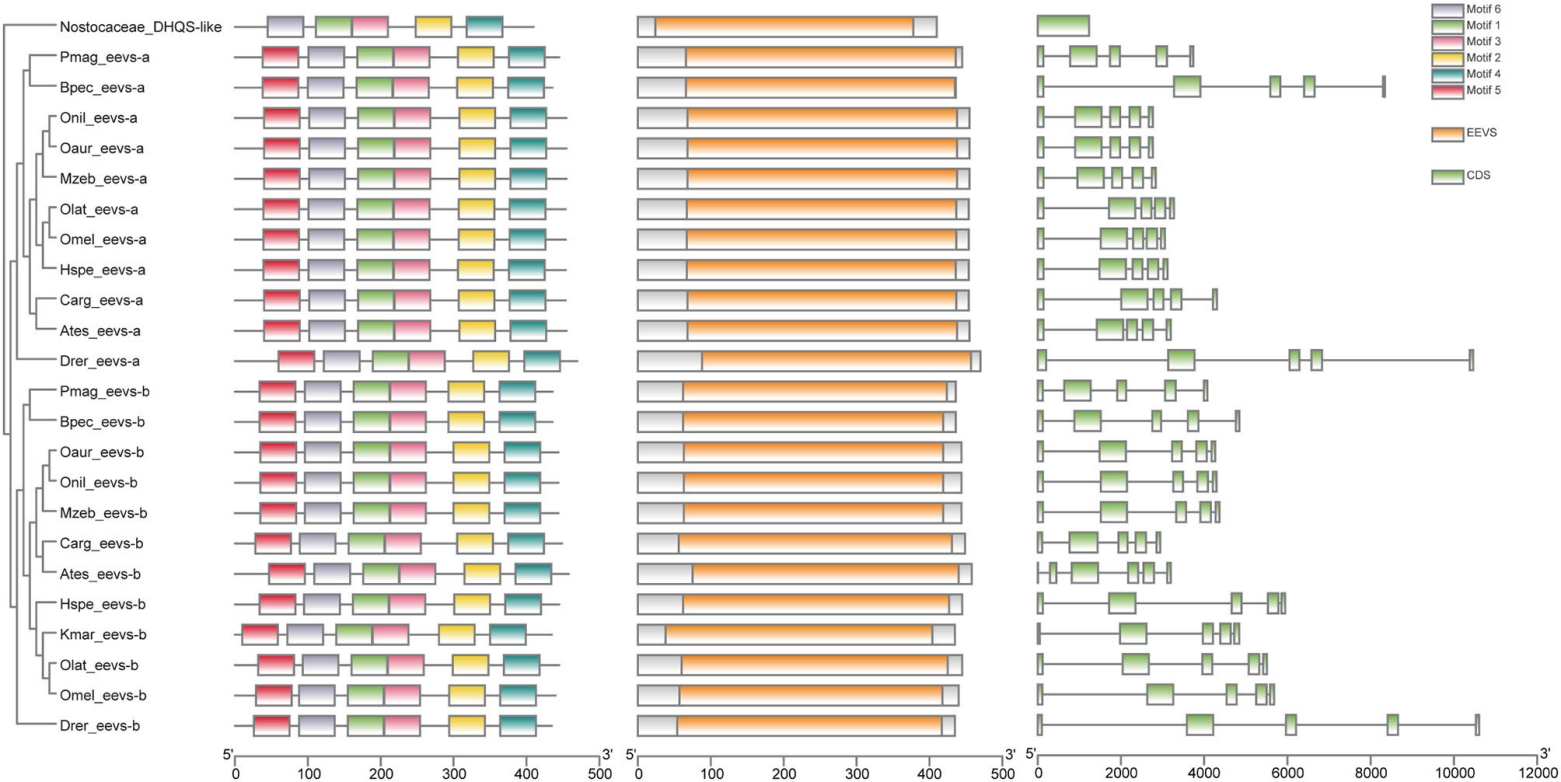
The rooted NJ tree of teleost *eevs*-like genes. It was constructed with cyanobacteria DHQS-like as the outgroup. The first column is the rooted tree, the second column is the six motifs derived from MEME web service, the third column is the conserved domain CDD derived from NCBI, and the four column is the detailed structures of *eevs*-like genes.

Low retinal dopamine levels could cause myopia (Feldkaemper and Schaeffel, 2013), and AANAT1a can reduce the dopamine content in the retina via acetylation (Zilberman-Peled et al., 2006). The loss of *aanat1a* in amphibious giant-fin mudskipper could be beneficial for movement in air (You et al., 2014). Interestingly, 12 teleost fishes except for giant-fin mudskipper have one copy of *annat1a* (see more details in Table 4; Figure 3). A previous study reported that the Atlantic flyingfish (*C. heterurus*) had a pyramidal shape cornea, which could assure both hypermetropic underwater vision and emmetropic vision in air (Baylor, 1967). Since the mirrorwing flyingfish owned three copies of *aanat* (without absence of *aanat1a*), its unique cornea might be responsible for a temporary air vision. Gadusol biosynthesis genes in the mirrorwing flyingfish we identify two copies of *eevs*-like and one copy of *mt-ox* in all the selected 12 fish genomes. Interestingly, the mirrorwing flyingfish has the same gene cluster as medaka, with *mdfic2* missing in the gene cluster “*foxp1b*-*mdfic2*-*mt-ox*-*eevs-a*-*mitfa*-*frmd4Ba*” (see more details in Table 5). All fishes shared the gene cluster of “*foxp1a*-*eevs-b*-*mitfb*-*frmd4Bb*” except for zebrafish (Supplementary Figure 11). Perhaps, the examined zebrafish genome was modified by genetic engineering (Carpio and Estrada, 2006). The two isotypes of *eevs*-like gene contain five exons, conserved domain CCD, and six conserved motifs (Figure 4). It seems that this Beloniformes species had experienced the same gene loss event.

**Table 4:** Copy number of *aanat* genes in the 12 representative teleost fishes.

**Species**	**Common Name**	**Total Number**	***aanat1a***	***aanat1b***	***aanat2***
*A. testudineus*	Climbing perch	3	1	1	1
*B. pectinirostris*	Blue-spotted mudskipper	3	1	1	1
*P. magnuspinnatus*	Giant-fin mudskipper	2	-	1	1
*C. argus*	Northern snakehead	3	1	1	1
*H. speculiger*	Mirrorwing flyingfish	3	1	1	1
*K. marmoratus*	Mangrove rivulus	3	1	1	1
*O. aureus*	Blue tilapia	3	1	1	1
*O. niloticus*	Nile tilapia	3	1	1	1
*M. zebra*	Zebra mbuna	3	1	1	1
*O. latipes*	Japanese medaka	3	1	1	1
*O. melastigma*	Indian medaka	3	1	1	1
*D. rerio*	Zebrafish	2	1	-	1

**Table 5:** Genetic analysis of *eevs* and *mt-ox* genes in selected fishes.

**Species**	**Common Name**	***foxp1bfoxp1a***	***mdfic2***	***mt-ox***	***eevsaeevsb***	***mitfamitfb***	***frmd4Bafrmd4Bb***
*A. testudineus*	Climbing perch	√√	√_2_×	√_2_×	√√	√√	√√
*B. pectinirostris*	Blue-spotted mudskipper	√√	√ ×	√ ×	√√	√√	√√
*P. magnuspinnatus*	Giant-fin mudskipper	√√	√ ×	√ ×	√√	√√	√√
*C. argus*	Northern snakehead	√√	√ ×	√ ×	√√	√√	√√
*H. speculiger*	Mirrorwing flyingfish	√√	× ×	√ ×	√√	√√	√√
*K. marmoratus*	Mangrove rivulus	√√	√ ×	√ ×	×√	√√	√√
*O. aureus*	Blue tilapia	√√	√ ×	√ ×	√√	√√	√√
*O. niloticus*	Nile tilapia	√√	√ ×	√ ×	√√	√√	√√
*M. zebra*	zebra mbuna	√√	√ ×	√ ×	√√	√√	√√
*O. latipes*	Japanese medaka	√√	× ×	√ ×	√√	√√	√√
*O. melastigma*	Indian medaka	√√	× ×	√ ×	√√	√√	√√
*D. rerio*	Zebrafish	√ ×	√ ×	√ ×	√√	√√	√√

*The √_2_ means the climbing perch has two mdfic2 and mt-ox in the gene cluster as follows: foxp1b-mdfic2-mt-ox-mdfic2-mt-ox-eevs-a-mitfa-frmd4Ba; However, zebrafish doesn't have the following gene cluster: foxp1a-eevs-b-mitfb-frmd4Bb. More details are provided in Supplementary Figure 11*.

### Olfactory Genes in the Mirrorwing Flyingfish

Olfaction is an essential component of the animal sensory system for perceiving water- and air-soluble chemicals that can help to localize food, predators, and spawning migration sites (Hopfield, 1991). We identified 781 intact OR genes in nine representative fishes (Supplementary Table 15). These identified ORs could be classified into five subfamilies, including delta, epsilon, zeta, eta, and beta (see more details in Supplementary Figure 12).

The mirrorwing flyingfish possessed 50 intact OR genes; among them, the number of air-/waterborne OR genes were much less than climbing perch, northern snakehead, and zebrafish. Surprisedly, we could not find any airborne OR gene in the mirrorwing flyingfish genome. Although this fish could glide a while above water, the detailed classification and copy numbers of OR genes appear to be the same as those in medaka, while they are different from amphibious fishes (such as mudskippers; see You et al., 2014).

## Conclusions

We obtained a draft genome assembly for the representative mirrorwing flyingfish with a hybrid method after Illumina and PacBio sequencing. We constructed a phylogenetic tree to illuminate the relationship of the mirrorwing flyingfish and other 18 teleost fishes. We also investigated vision-related genes, olfactory receptor genes, and gadusol synthesis-related genes in representative teleost fishes. Since the mirrorwing flyingfish could leave water for a while, it may exhibit similar traits as amphibious fishes. However, our genomic comparisons of vision-related and olfactory receptor genes revealed that the mirrorwing flyingfish potentially shared the same genetic mechanisms as its phylogenetic relatives (medaka species) but different from popular amphibious fishes (such as mudskippers). This high-quality genome assembly provides a valuable genetic resource for the mirrorwing flyingfish, and it will also facilitate in-depth biomedical studies on various Exocoetoidea fishes.

## Data Availability Statement

The datasets presented in this study can be found in online repositories. The names of the repository/repositories and accession number(s) can be found below: https://www.ncbi.nlm.nih.gov/genbank/, PRJNA714815; https://figshare.com/, https://doi.org/10.6084/m9.figshare.14600634.v1.

## Ethics Statement

The animal study was reviewed and approved by Animal Care and Use Committee of BGI (approval ID: FT18134).

## Author Contributions

QS conceived the project. PX, CZ, CB, and XY analyzed the data. XY, JC, ZR, FY, RG, and JX collected samples and assisted data analysis. PX and CZ wrote the manuscript. QS and CB revised the manuscript. All authors approved submission of the final manuscript for publication.

## Conflict of Interest

The authors declare that the research was conducted in the absence of any commercial or financial relationships that could be construed as a potential conflict of interest.

## Figures and Tables

**Table:** 

Ates,	*Anabas testudineus*
Bpec,	*Boleophthalmus pectinirostris*
Carg,	*Channa argus*
Drer,	*Danio rerio*
Kmar,	*Kryptolebias marmoratus*
Mzeb,	*Maylandia zebra*
Oaur,	*Oreochromis aureus*
Onil,	*Oreochromis niloticus*
Olat,	*Oryzias latipes*
Omel,	*Oryzias melastigma*
Pmag,	*Periophthalmus magnuspinnatus*
Hspe,	*Hirundichthys speculiger*
OR,	olfactory receptor
AANAT,	aralkylamine N-acetyltransferase
TNPO3,	transportin 3
CALUA,	calumenin
SOCS2,	cytochrome c oxidase assembly protein
IRF5,	interferon regulatory factor 5
SWS1,	short wavelength-sensitive 1
HCFC1,	host cell factor C1
LWS,	long wavelength-sensitive
SWS2,	short wavelength-sensitive 2
TFE3b,	transcription factor binding to IGHM enhancer 3
GNL3L,	guanine nucleotide binding protein-like 3-like
SLC6A22.2,	solute carrier family 6 member 22, tandem duplicate 2
RH2,	green-sensitive
SLC6A22.1,	solute carrier family 6 member 22, tandem duplicate 1
SYNPR,	synaptoporin
PRICKLE2,	prickle homolog 2
RH1,	rhodopsin
ADAMTS9,	ADAM metallopeptidase with thrombospondin type 1 motif 9
MAGI1,	membrane-associated guanylate kinase, WW and PDZ domain containing 1
FRMD4B,	FERM domain containing 4B
MDFIC2,	MyoD family inhibitor domain-containing protein 2
FOXP1,	forkhead box P1
MITFA,	melanocyte inducing transcription factor a
MITFB,	melanocyte inducing transcription factor b
EEVS,	2-epi-5-epi-valiolone synthase
MT-Ox,	S-adenosyl-L-methionine-dependent methyltransferase
IRF10,	interferon regulatory factor 10
ATAXIN1,	ataxin-1
RAB32,	Ras-related protein Rab-32
STXBP5B,	syntaxin-binding protein 5b (tomosyn)
SASH1,	SAM and SH3 domain-containing protein 1

## Supplementary Material

Supplementary Figure 1 Pipeline of the genome assembly.

Supplementary Figure 2 Distribution of divergence rates in each type of TEs within the mirrorwing flyingfish genome. The divergence rate was calculated between the identified TEs in the genome and the consensus sequences in the TE library used Repbase **(A)** or *de novo* libraries constructed by RepeatModeler and LTR_Finder **(B)**.

Supplementary Figure 3 Evolution of 19 representative teleost genomes and their gene families. **(A)** Divergence tree with expanded and contracted gene families. **(B)** Statistics of single-copy orthologs, multiple-copy orthologs, unique paralogs, other orthologs, and unclustered gene numbers in the 19 teleost fishes.

Supplementary Figure 4 A rooted phylogenetic tree (constructed with Bayes).

Supplementary Figure 5 A rooted phylogenetic tree (constructed with PhyML).

Supplementary Figure 6 A fossil-calibrated phylogenetic tree. It was constructed with the following five calibrated times that were adapted from the Timetree: *C. argus*-*A. testudineus* (66~78Mya), *H. speculiger*-*O. latipes* (68~89 Mya), *O. latipes*-*O. aureus* (104~145 Mya), *B. pectinirostris*-*P. magnuspinnatus* (49~69 Mya), and *D. rerio***-***O. aureus* (149~165 Mya).

Supplementary Figure 7 The crucial tuning sites in LWS opsins of 12 teleost fishes and human. Five critical sites in the mirrorwing flyingfish include S180A, H197Y, Y277F, T285A, and A308S. Abbreviations of fish species are provided in Supplementary Table 1.

Supplementary Figure 8 The gene architecture of *RH1* **(A)** and *SWS1* **(B)** in 12 representative teleost fishes. Gene names were listed in the first line. Abbreviations of fish species are provided in Supplementary Table 1.

Supplementary Figure 9 The gene architecture of *LWS*-*SWS2* **(A)** and *RH2* **(B)** in 12 representative teleost fishes. Gene names were listed in the first line. Abbreviations of fish species are provided in Supplementary Table 1.

Supplementary Figure 10 The crucial tuning site of SWS1 (F86V) in 8 selected teleost fishes. The tuning site was marked in light blue. Abbreviations of fish species are provided in Supplementary Table 1.

Supplementary Figure 11 The gene architecture of *eevs*-likes and *mt-ox* in12 representative teleost fishes. Gene names were listed in the first line. Abbreviations of fish species are provided in Supplementary Table 1.

Supplementary Figure 12 A rooted neighbor-joining tree of olfactory receptor genes (ORs) in 9 selected teleost fishes. A total of 787 sequences were collected for construction of the circular cladogram tree. Various OR types were marked in different colors.

Supplementary Table 1 Information of 19 teleost fishes used in our present study.

Supplementary Table 2 Accession numbers of known aanat, opsin and neighboring genes.

Supplementary Table 3 Accession numbers of known adjacent genes of eevs and mt-ox.

Supplementary Table 4 Libraries and data yields for the whole genome shotgun sequencing.

Supplementary Table 5 Libraries and data yields for the PacBio sequencing.

Supplementary Table 6 The alignment result of paired-end reads mapping to H. speculiger genome.

Supplementary Table 7 Summary of repeat annotations.

Supplementary Table 8 Classification of repetitive elements.

Supplementary Table 9 Statistics of function annotation.

Supplementary Table 10 Statistics of Non-coding RNAs in the genome.

Supplementary Table 11 Statistics of gene families in 19 teleost fishes.

Supplementary Table 12 Statistics of pairwise alignment among mirrorwing flyingfish, medaka and zebrafish.

Supplementary Table 13 KEGG enrichment for genes of expanded gene families of the mirrorwing flyingfish.

Supplementary Table 14 Estimated maximal absorption spectrum (?max) of LWS.

Supplementary Table 15 Copy numbers of intact OR genes in each group of fishes and mammals.
