# Supplementary figures and images for: Draft Genome of the Mirrorwing Flyingfish (Hirundichthys speculiger)
Source: Front Genet. 2021 Jul 7;12:695700. doi: 10.3389/fgene.2021.695700 (PMC8294118; doi:10.3389/fgene.2021.695700)

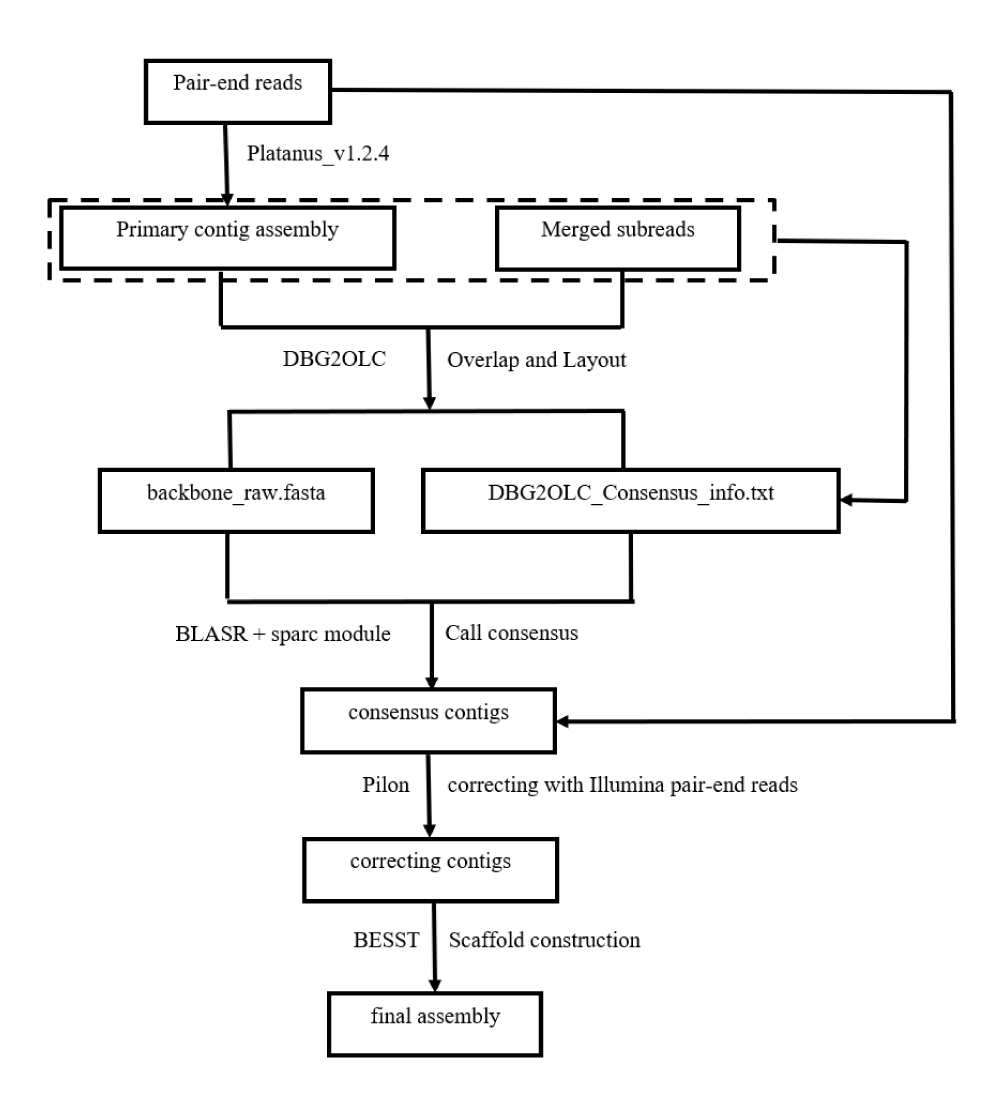

Supplement: Supplementary Figure 1 — Pipeline of the genome assembly. [file Data_Sheet_1.ZIP › Supplementary Figure 1.tif]

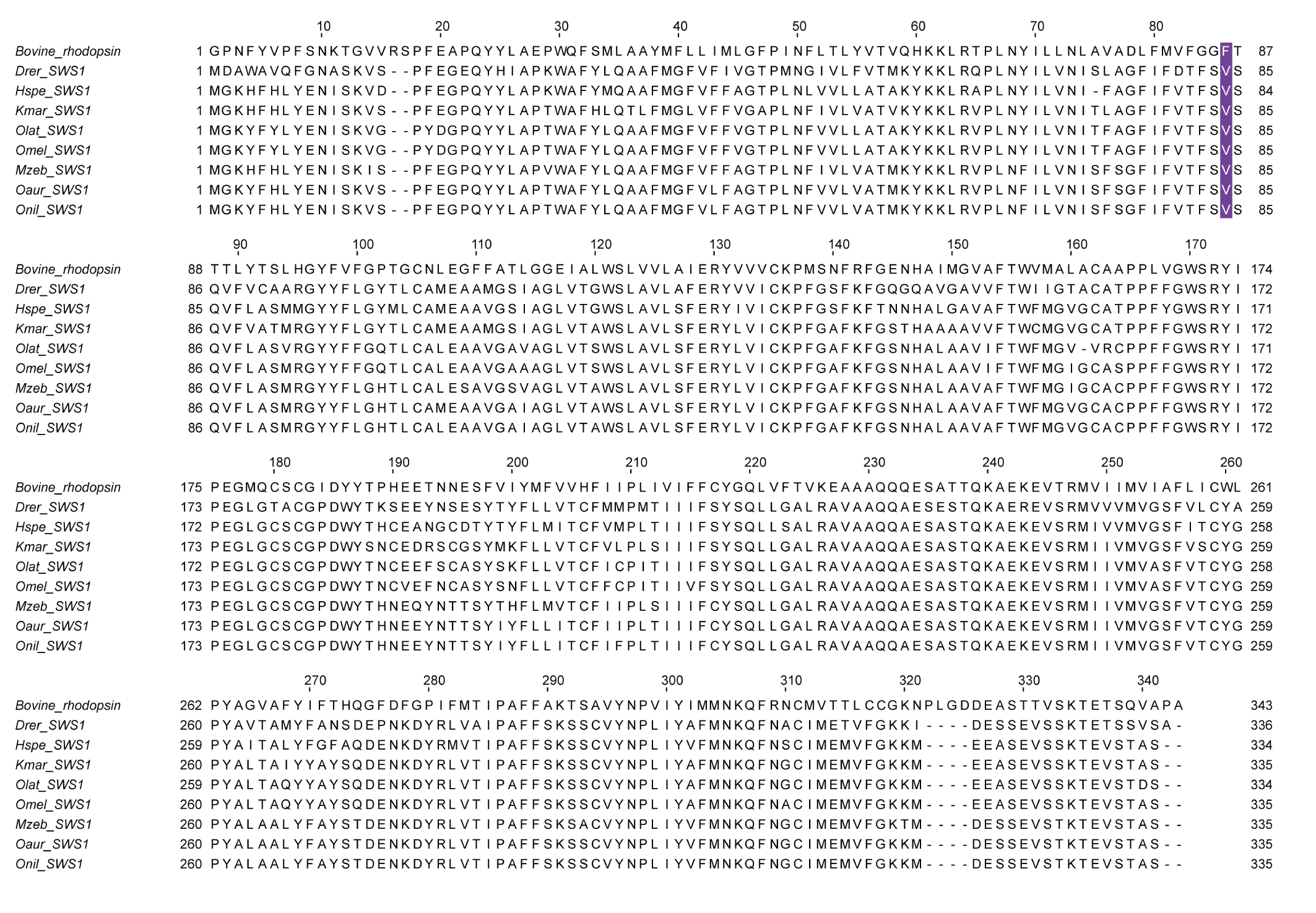

Supplement: Supplementary Figure 1 — Pipeline of the genome assembly. [file Data_Sheet_1.ZIP › Supplementary Figure 10.tif]

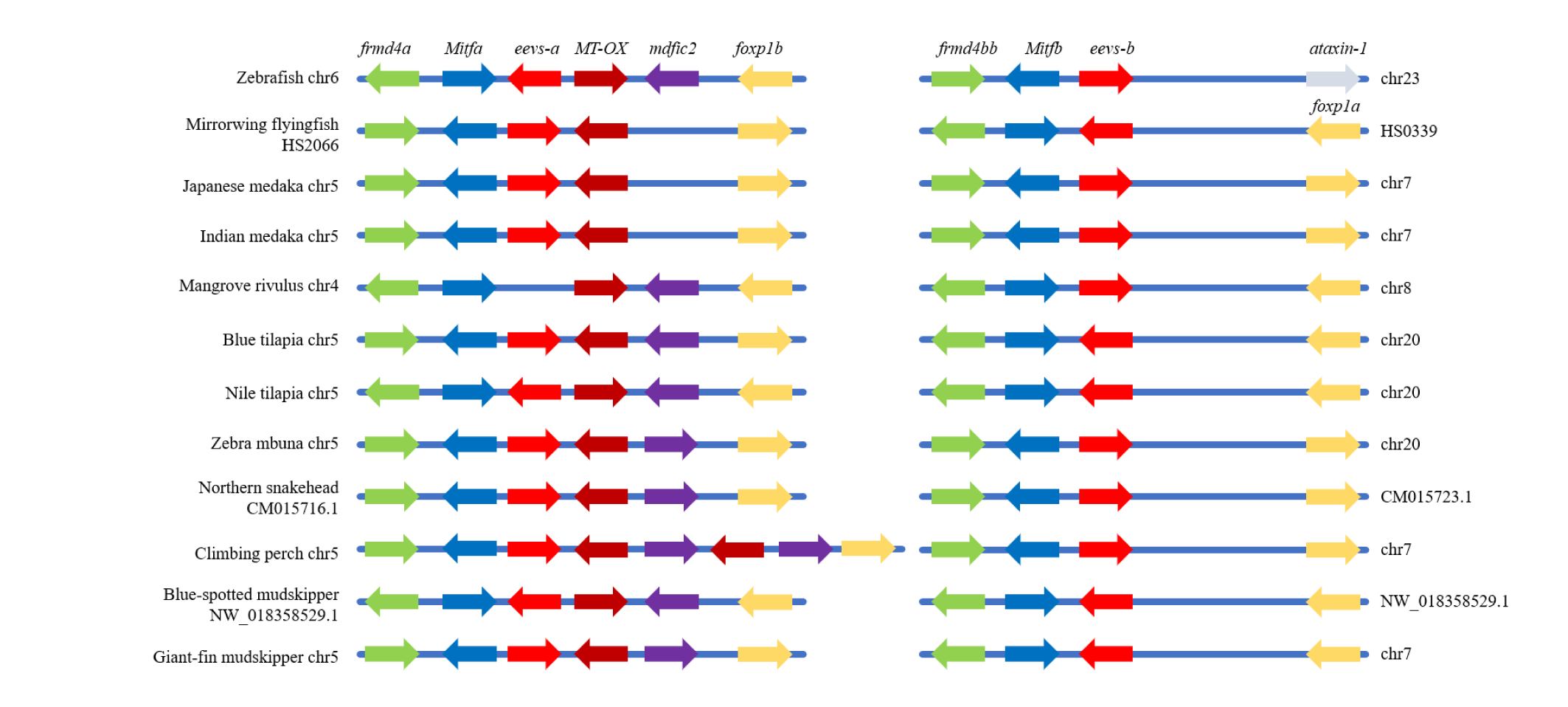

Supplement: Supplementary Figure 1 — Pipeline of the genome assembly. [file Data_Sheet_1.ZIP › Supplementary Figure 11.tif]

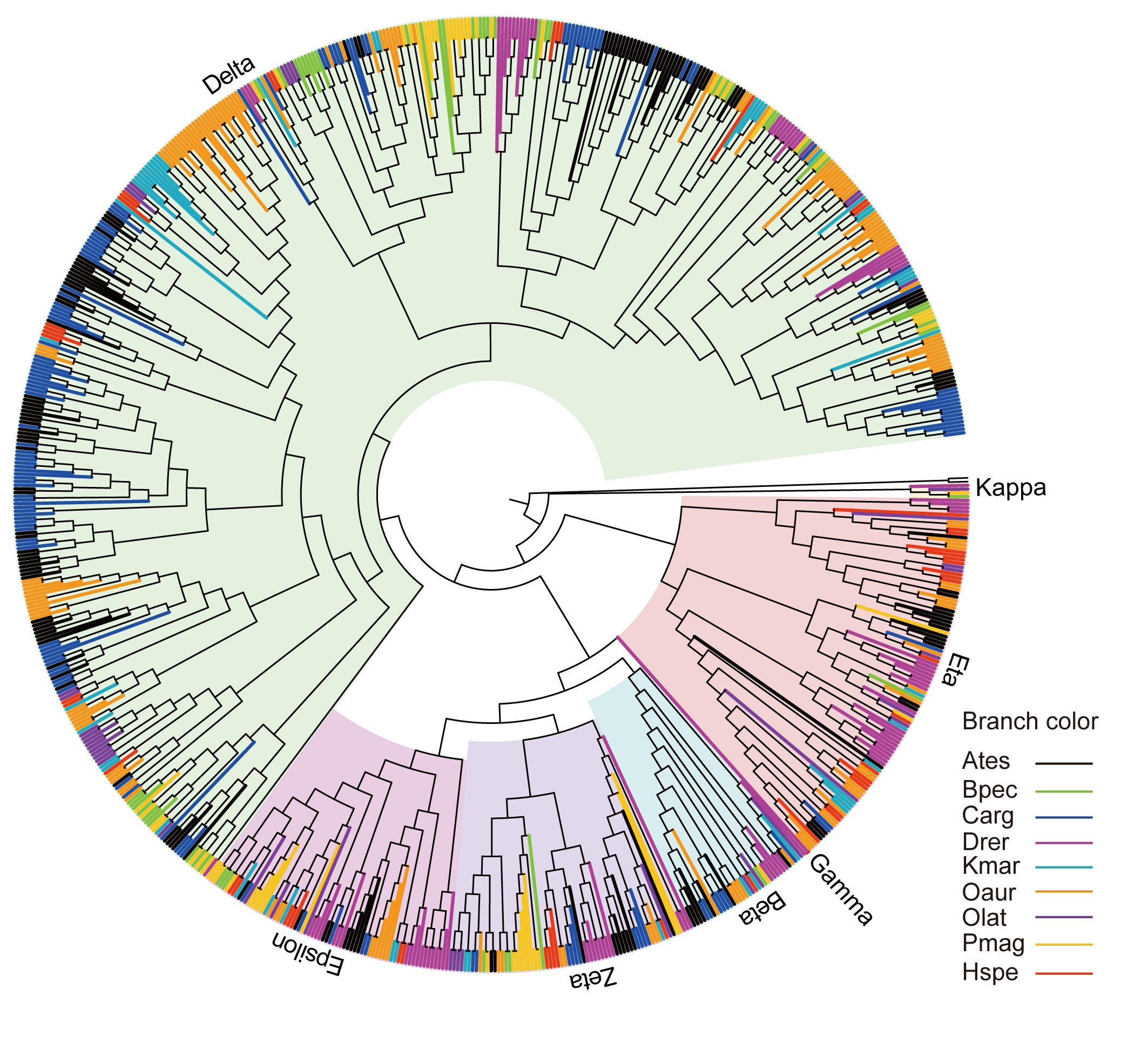

Supplement: Supplementary Figure 1 — Pipeline of the genome assembly. [file Data_Sheet_1.ZIP › Supplementary Figure 12.tif]

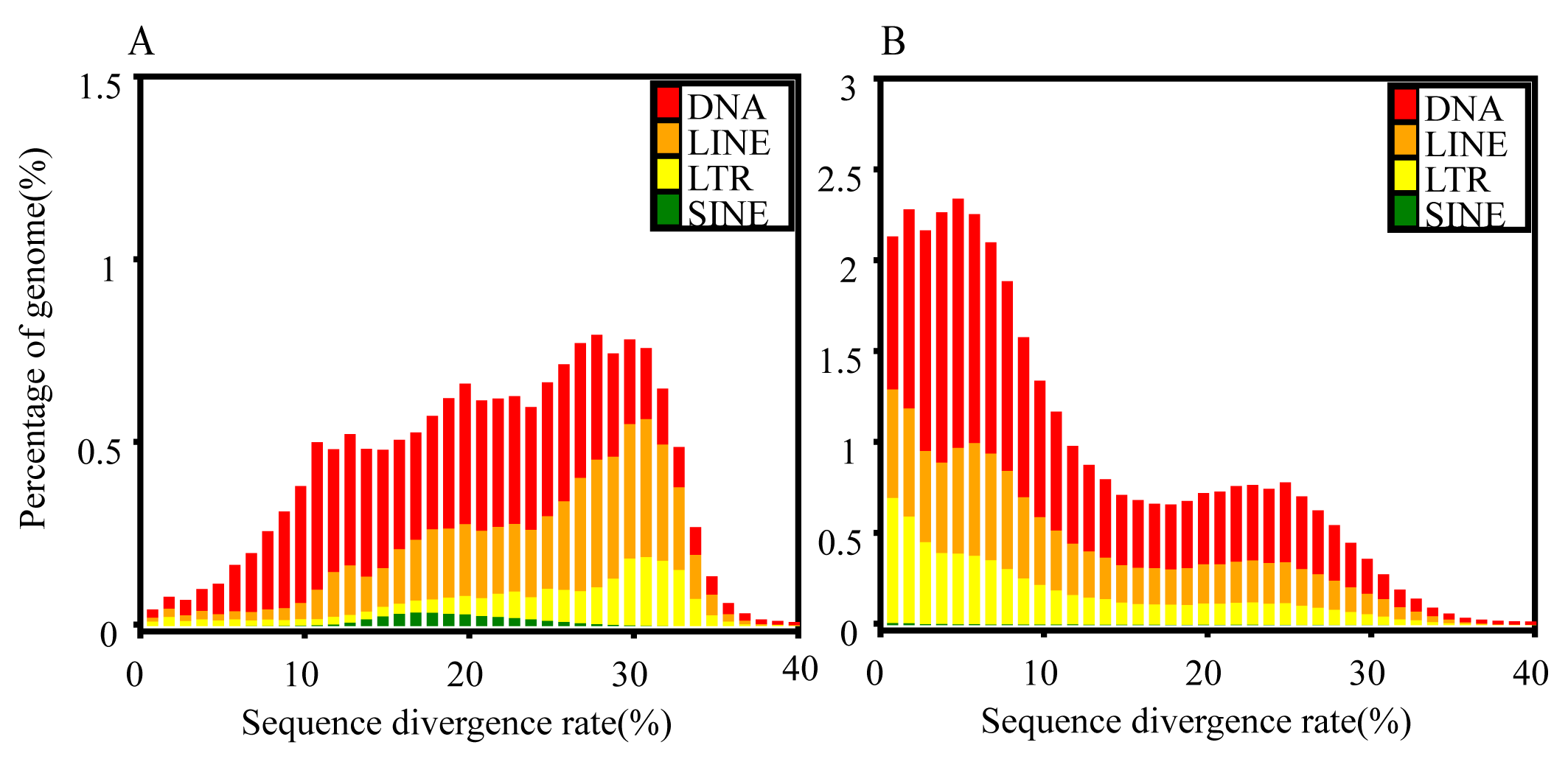

Supplement: Supplementary Figure 1 — Pipeline of the genome assembly. [file Data_Sheet_1.ZIP › Supplementary Figure 2.tif]

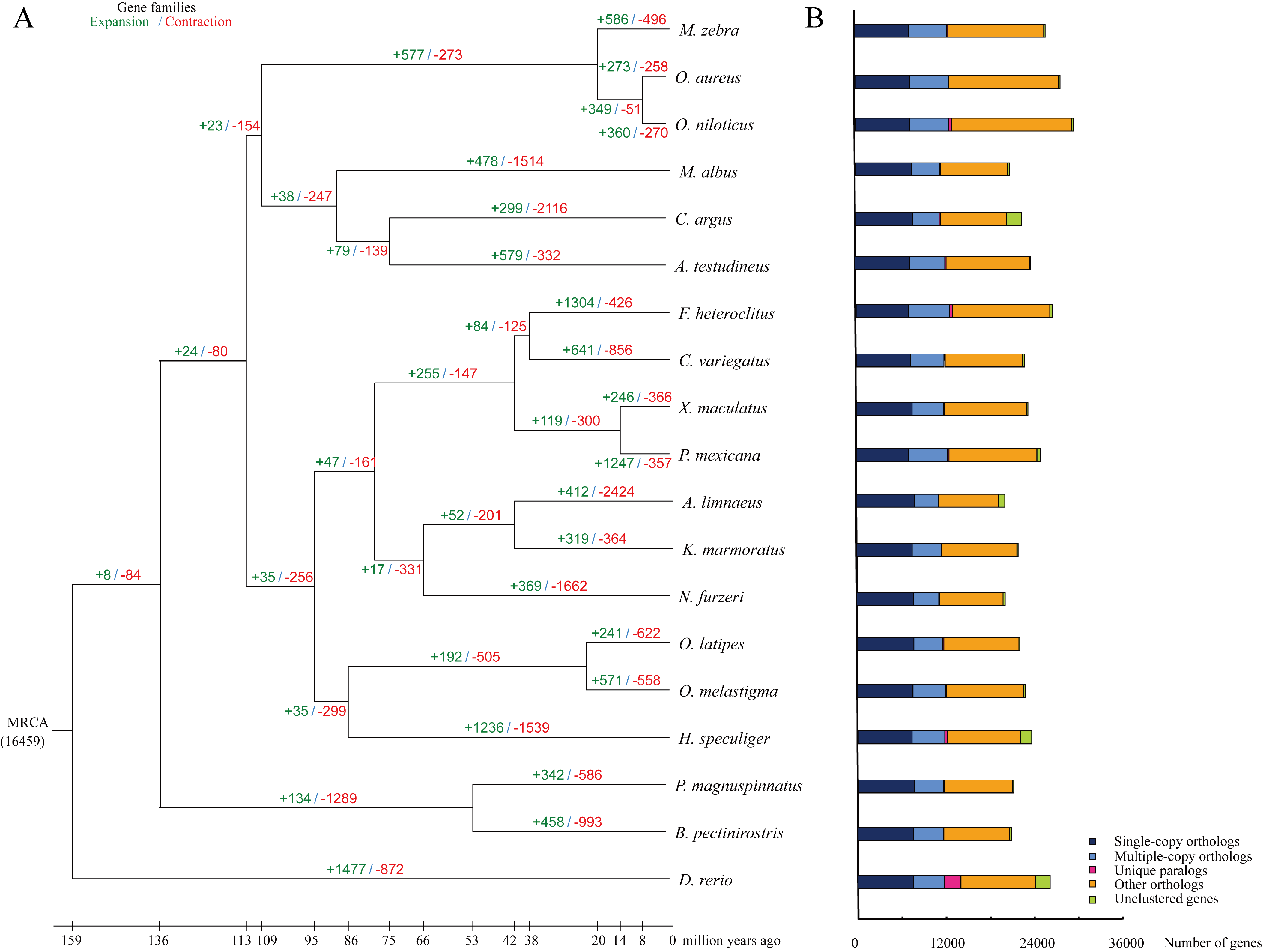

Supplement: Supplementary Figure 1 — Pipeline of the genome assembly. [file Data_Sheet_1.ZIP › Supplementary Figure 3.tif]

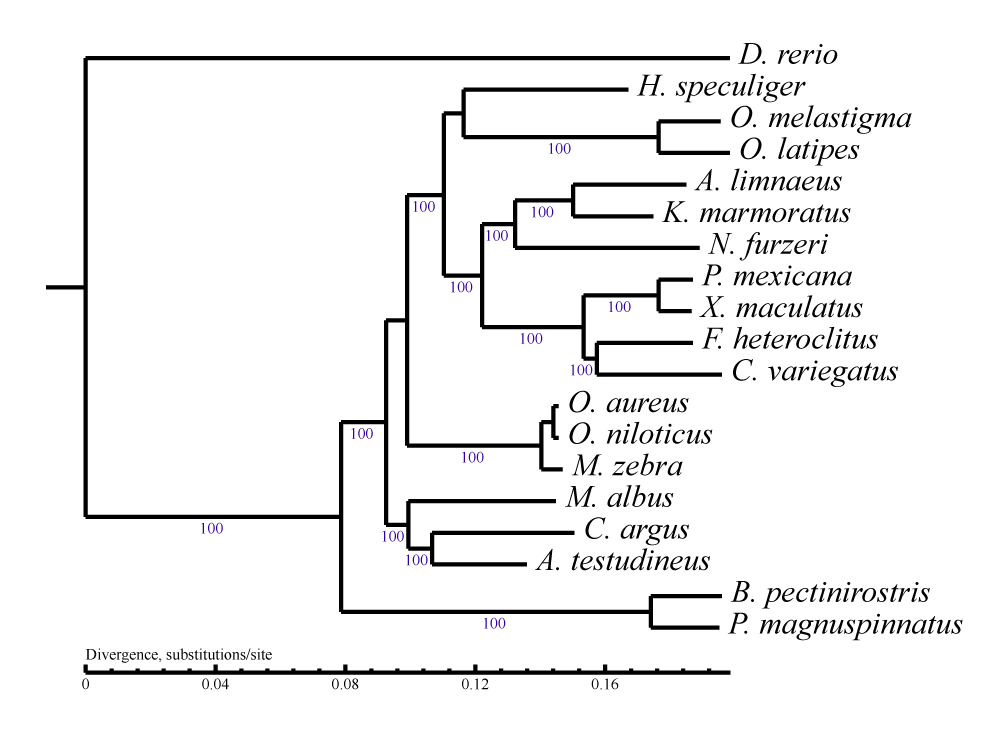

Supplement: Supplementary Figure 1 — Pipeline of the genome assembly. [file Data_Sheet_1.ZIP › Supplementary Figure 4.tif]

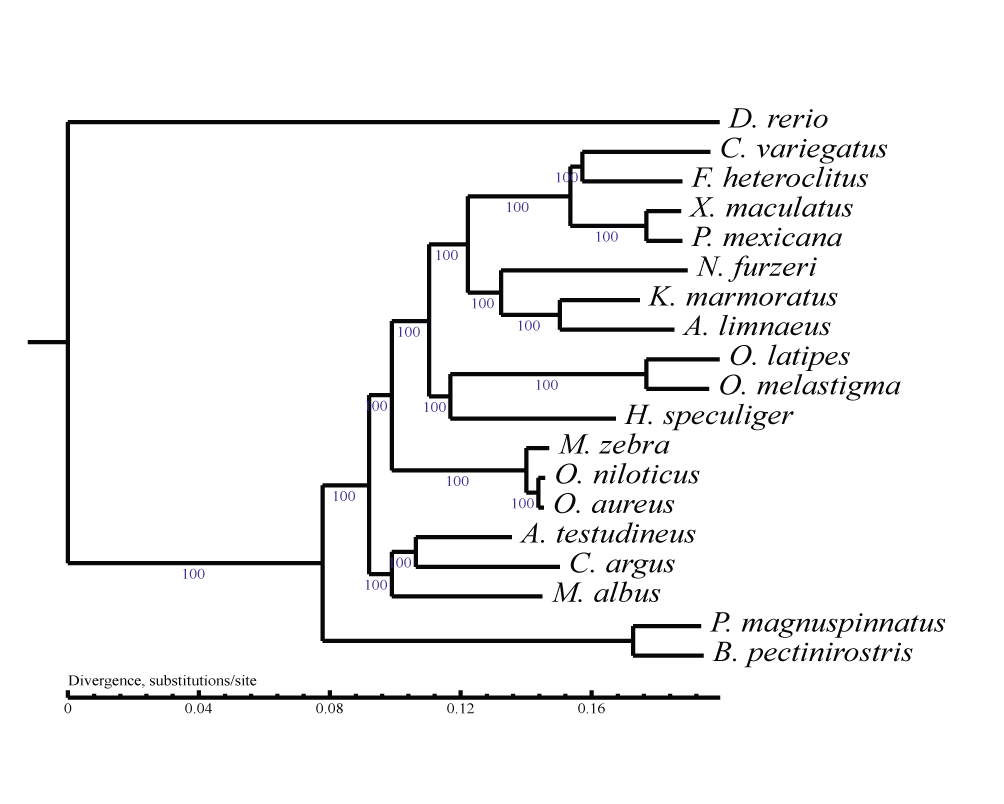

Supplement: Supplementary Figure 1 — Pipeline of the genome assembly. [file Data_Sheet_1.ZIP › Supplementary Figure 5.tif]

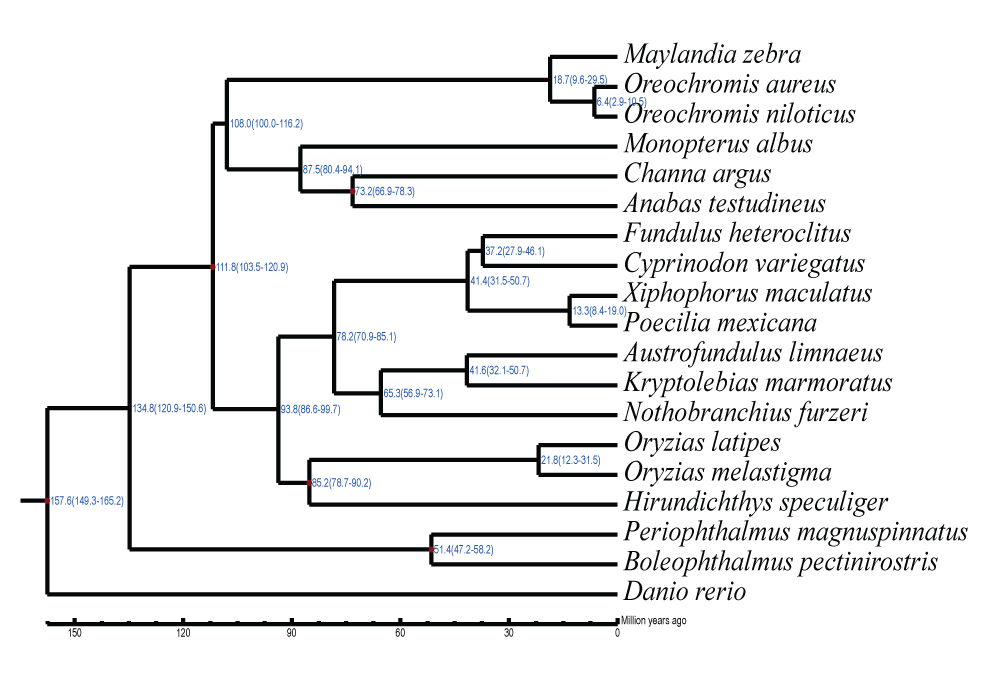

Supplement: Supplementary Figure 1 — Pipeline of the genome assembly. [file Data_Sheet_1.ZIP › Supplementary Figure 6.tif]

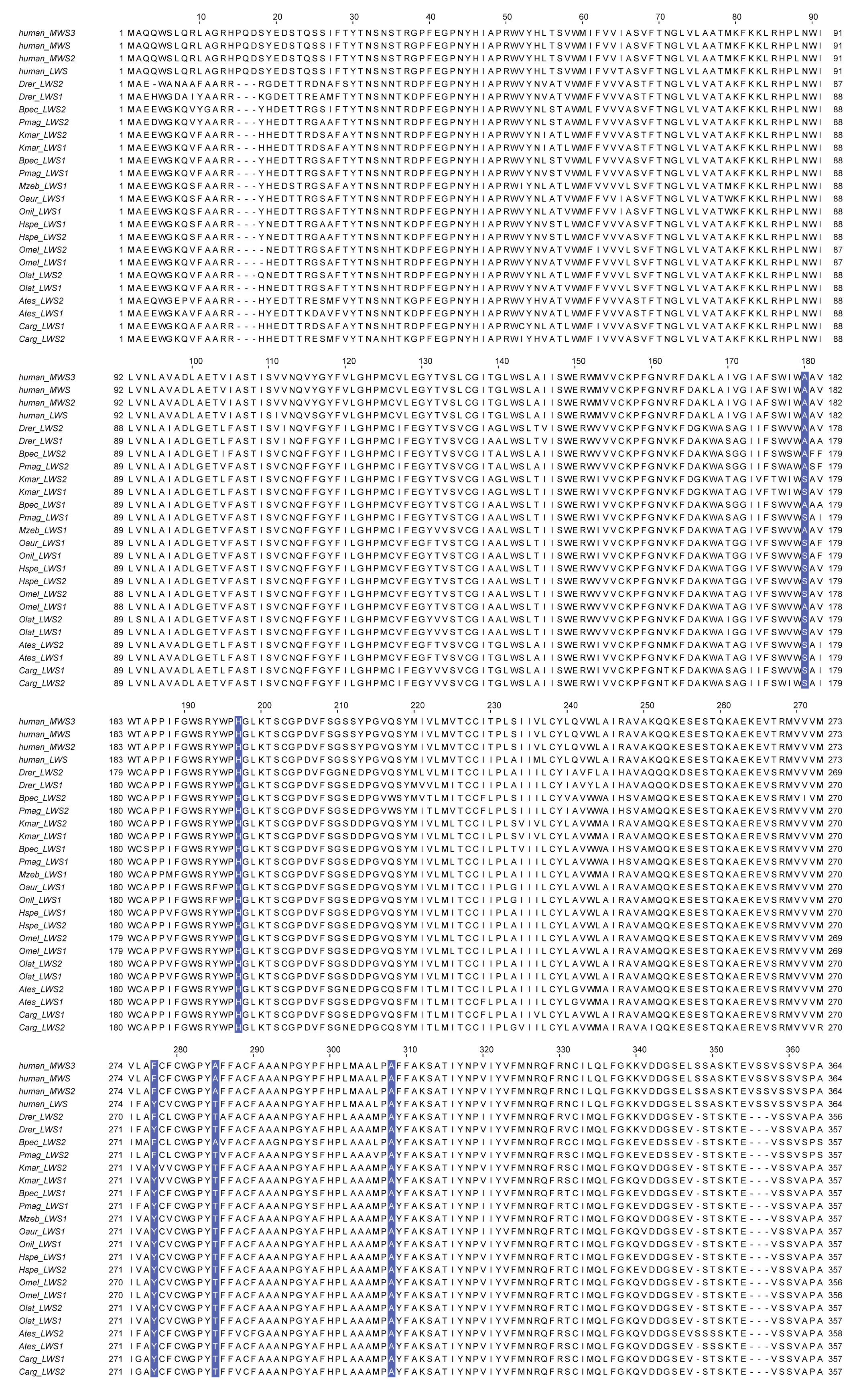

Supplement: Supplementary Figure 1 — Pipeline of the genome assembly. [file Data_Sheet_1.ZIP › Supplementary Figure 7.tif]

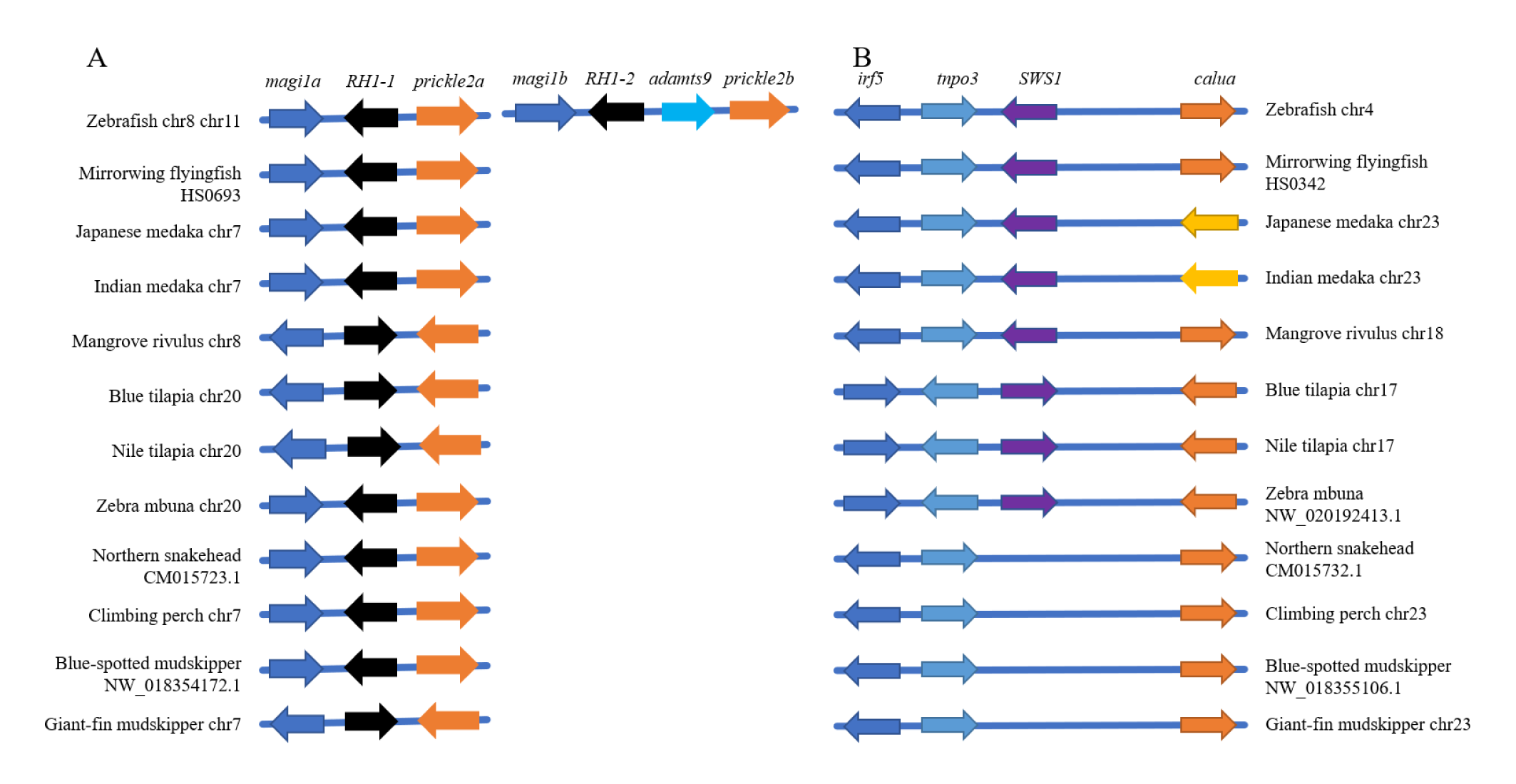

Supplement: Supplementary Figure 1 — Pipeline of the genome assembly. [file Data_Sheet_1.ZIP › Supplementary Figure 8.tif]

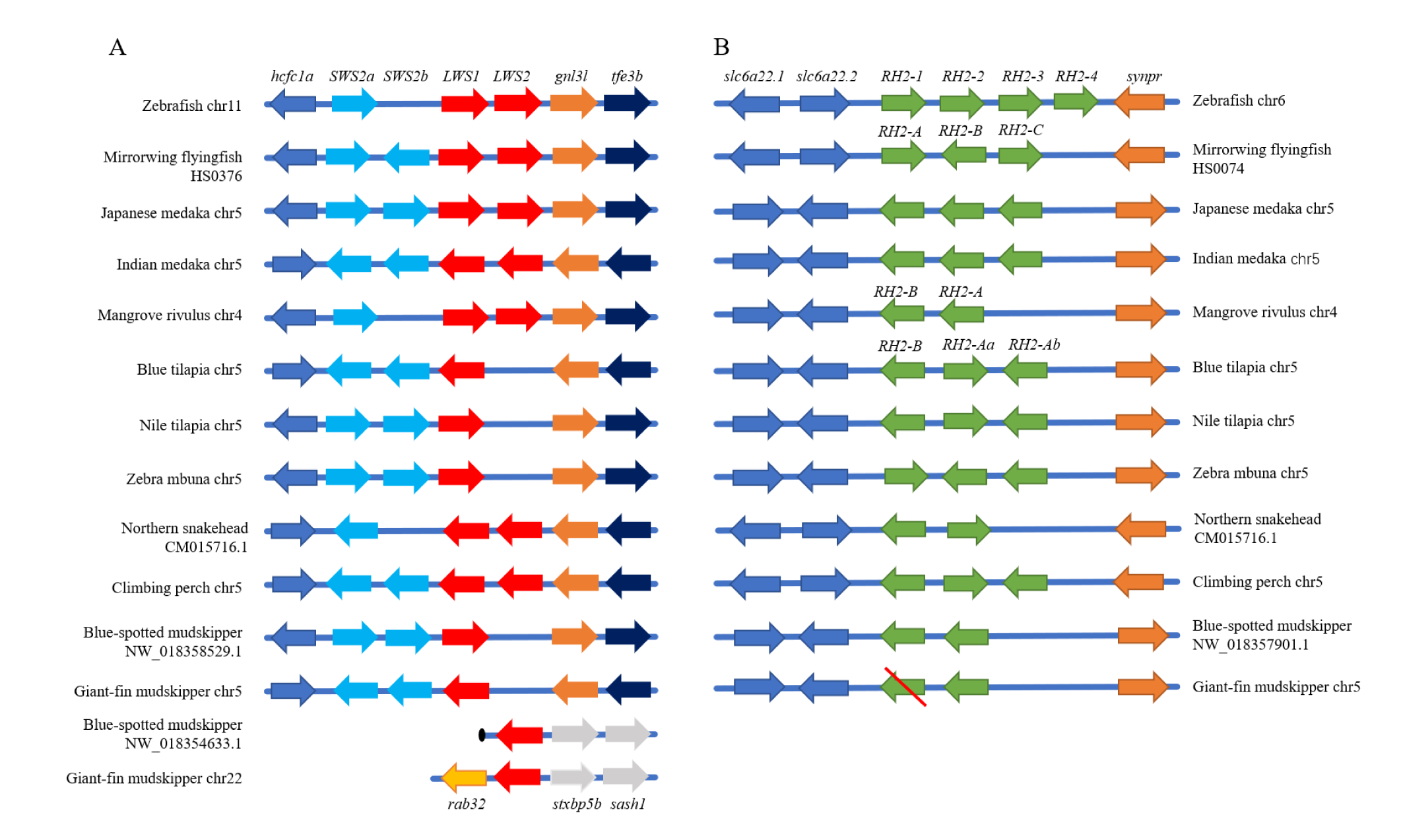

Supplement: Supplementary Figure 1 — Pipeline of the genome assembly. [file Data_Sheet_1.ZIP › Supplementary Figure 9.tif]
