# Supplementary material for: Draft Genome of the Mirrorwing Flyingfish (Hirundichthys speculiger)
Source: Front Genet. 2021 Jul 7;12:695700. doi: 10.3389/fgene.2021.695700 (PMC8294118; doi:10.3389/fgene.2021.695700)
Supplement: Supplementary Table 1 — Information of 19 teleost fishes used in our present study. [file Data_Sheet_2.docx]

**Supplementary Table 1** Information of 19 teleost fishes used in our present study

| Species | Common name | NCBI Source | Abbreviation |
| --- | --- | --- | --- |
| *Anabas testudineus* | Climbing perch | GCF_900324465.2 | Ates |
| *Austrofundulus limnaeus* | Annual killifish | GCF_001266775.1 |  |
| *Boleophthalmus pectinirostris* | Blue-spotted mudskipper | GCF_000788275.1 | Bpec |
| *Channa argus* | Northern snakehead | GCA_004786185.1 | Carg |
| *Cyprinodon variegatus* | Sheepshead minnow | GCF_000732505.1 |  |
| *Danio rerio* | Zebrafish | GCF_000002035.6 | Drer |
| *Fundulus heteroclitus* | Mummichog | GCF_011125445.2 |  |
| *Kryptolebias marmoratus* | Mangrove rivulus | GCF_001649575.2 | Kmar |
| *Maylandia zebra* | Zebra mbuna | GCF_000238955.4 | Mzeb |
| Monopterus albus | Asian swamp eel | GCF_001952655.1 |  |
| Nothobranchius furzeri | Turquoise killifish | GCA_014300015.1 |  |
| *Oreochromis aureus* | Blue tilapia | GCF_013358895.1 | Oaur |
| *Oreochromis niloticus* | Nile tilapia | GCF_001858045.2 | Onil |
| *Oryzias latipes* | Japanese medaka | GCF_002234675.1 | Olat |
| *Oryzias melastigma* | Indian medaka | GCF_002922805.2 | Omel |
| *Periophthalmus magnuspinnatus* | Giant-fin mudskipper | GCF_009829125.1 | Pmag |
| *Poecilia Mexicana* | Shortfin molly | GCF_001443325.1 |  |
| *Xiphophorus maculatus* | Southern platyfish | GCF_002775205.1 |  |
| *Hirundichthys speculiger* | Mirrorwing flyingfish | this study assembly | Hspe |

**Supplementary Table 2** Accession numbers of known *aanat*, opsin and neighboring genes

| Abbreviation | Protein accession | | |
| --- | --- | --- | --- |
|  | *Danio rerio* | *Oreochromis aureus* | *Oryzias latipes* |
| AANAT1a | NP_956998.1 | XP_031583591.1 | XP_020559986.1 |
| AANAT1b | - | XP_031604511.1 | XP_011476720.1 |
| AANAT2 | NP_571486.1 | XP_031593256.1 | NP_001098316.1 |
| TFE3b | NP_001038531.1 | XP_031588876.2 | XP_020559128.1 |
| GNL3L | NP_001002875.1 | XP_031588877.1 | XP_004069143.1 |
| LWS2 | NP_001002443.1 | - | NP_001098164.1 |
| LWS1 | NP_001300644.1 | XP_031588878.1 | XP_004069142.1 |
| SWS2a | NP_571267.1 | XP_031588879.1 | XP_004069275.1 |
| SWS2b | - | XP_031588880.1 | NP_001098124.1 |
| HCFC1a | NP_001038529.1 | XP_031588883.1 | XP_011473718.1 |
| MAGI1a | XP_021334196.1 | XP_039460384.1 | XP_011475551.1 |
| RH1-1 | NP_571159.1 | XP_031609745.1 | NP_001098165.1 |
| PRICKLE2a | NP_001071251.2 | XP_031609467.1 | XP_004070576.1 |
| MAGI1b | XP_005155886.1 | - | - |
| RH1-2 | NP_001103501.1 | - | - |
| ADAMTS9 | NP_001244125.1 | - | - |
| PRICKLE2b | XP_005155884.1 | - | - |
| SYNPR | NP_001004532.1 | XP_031613516.1 | XP_004086218.1 |
| RH2 | NP_571329.1 | XP_031613515.1 | XP_004086180.1 |
|  | NP_878312.1 | XP_031613513.1 | XP_004086178.2 |
|  | NP_878311.1 | XP_031613514.1 | NP_001098125.1 |
|  | NP_571328.2 | - | - |
| SLC6A22.2 | XP_005166102.1 | XP_031613368.2 | XP_023810430.1 |
| SLC6A22.1 | XP_005166076.1 | XP_031613391.1 | XP_020557092.1 |
| IRF5 | NP_001314746.1 | XP_031587416.1 | XP_004085073.1 |
| TNPO3 | NP_957381.1 | XP_031587414.1 | XP_023807927.1 |
| SWS1 | NP_571394.1 | XP_031587413.1 | NP_001098126.1 |
| CALUA | NP_001002151.2 | XP_039457244.1 | XP_004084835.1(socs2) |

**Supplementary Table 3** Accession numbers of known adjacent genes of *eevs* and *mt-ox*

| Abbreviation | Protein accession | | |
| --- | --- | --- | --- |
|  | *Danio rerio* | *Oreochromis aureus* | *Oryzias latipes* |
| FOXP1b | XP_021332591.1 | XP_039468020.1 | XP_023810577.1 |
| MDFIC2 | XP_017211919.1 | XP_039468702.1 | - |
| MT-OX | NP_001013468.1 | XP_031585699.1 | XP_004068646.1 |
| EEVS-a | XP_001343422.1 | XP_031585692.1 | XP_004068647.3 |
| MITFA | NP_570998.1 | XP_039468712.1 | NP_001269217.1 |
| FRMD4Ba | XP_021332923.1 | XP_031587637.1 | XP_011473059.1 |
| FOXP1a | - | XP_039460748.1 | XP_023812517.1 |
| IRF10 | XP_005161951.1 | - | - |
| ATAXIN1 | XP_700412.6 | - | - |
| EEVS-b | XP_009294834.1 | XP_031583777.1 | XP_004070973.1 |
| MITFB | NP_571922.2 | XP_031583757.1 | XP_011475737.2 |
| FRMD4Bb | XP_009294836.1 | XP_031583759.1 | XP_023812523.1 |

**Supplementary Table 4** Libraries and data yields for the whole genome shotgun sequencing

| Library type | Lane | Platform | Insert  Size (bp) | Read Length (bp) | Raw  bases (Gb) | Clean  bases (Gb) |
| --- | --- | --- | --- | --- | --- | --- |
| PE150 | 1 | X-Ten | 270 | 150 | 32.40 | 29.79 |
| PE150 | 3 | X-Ten | 500 | 150 | 34.40 | 23.84 |
| PE125 | 1 | X-Ten | 800 | 125 | 27.17 | 23.08 |
| MP150 | 1 | X-Ten | 2,000 | 150 | 16.18 | 8.09 |
| MP150 | 1 | X-Ten | 5,000 | 150 | 16.97 | 8.29 |
| MP150 | 1 | X-Ten | 10,000 | 150 | 11.01 | 6.12 |
| Total | 8 |  |  |  | 138.13 | 99.21 |

Note: PE, paired-end; MP, mate pair. The raw reads were filtered using SOAPnuke with parameter “-n 0.02 -Q 2 -l 15 -5 1 -d -I -q 0.4”.

**Supplementary Table 5** Libraries and data yields for the PacBio sequencing

| ID | Total  bases (Gb) | Total  reads | Average  Length (bp) | Max  Length (bp) | Min  Length (bp) | N50  Length (bp) |
| --- | --- | --- | --- | --- | --- | --- |
| m54169_180205_133128 | 5.43 | 521,237 | 10,423 | 1,00,085 | 50 | 16,121 |
| m54169_180208_002542 | 5.91 | 565,862 | 10,447 | 1,11,487 | 50 | 16,053 |
| m54173_180201_205646 | 3.37 | 298,197 | 11,305 | 117,372 | 50 | 17,038 |
| m54173_180203_052256 | 5.08 | 460,828 | 11,013 | 65,089 | 50 | 17,048 |
| m54173_180203_152939 | 4.76 | 438,864 | 10,851 | 83,330 | 50 | 16,444 |
| m54173_180204_013918 | 5.43 | 500,356 | 10,860 | 178,428 | 50 | 16,651 |
| Merge subreads | 29.98 | 2,785,344 | 10,766 | 178,428 | 50 | 16,510 |
| Correct with Mecat | 15.52 | 1,677,589 | 9,254 | 57,846 | 2,000 | 11,940 |

Note: the subreads were generated by smrtlink_4.0.0.190159 with bam2fasta.

**Supplementary Table 6** The alignment result of paired-end reads mapping to *H. speculiger* genome

| Category | of genome (%) |
| --- | --- |
| Paired mapping rate (%) | 94.91 |
| Coverage (%) | 97.78 |
| Coverage at least 10 X (%) | 97.76 |
| Coverage at least 20X (%) | 97.58 |

Note: Mapping rate: The number of total reads that mapped to the reference genome. Coverage: The sequence coverage of the reference genome. Coverage at least 10X: The percentage of bases with depth >10X in whole genome bases. Coverage at least 20X: The percentage of bases with depth >20X in whole genome bases.

**Supplementary Table 7** Summary of repeat annotations

| Type | Repeat Size (bp) | % of genome |
| --- | --- | --- |
| Trf | 85,463,920 | 8.193681 |
| Repeatmasker | 149,259,513 | 14.309954 |
| Proteinmask | 63,960,271 | 6.132062 |
| De novo | 436,759,339 | 41.873419 |
| Total | 483,328,548 | 46.338148 |

**Supplementary Table 8** Classification of repetitive elements

| Category | RepBase TEs | | TE Proteins | | De novo TE | | Combined TEs | |
| --- | --- | --- | --- | --- | --- | --- | --- | --- |
|  | Length  (bp) | Percentage  (%) | Length  (bp) | Percentage  (%) | Length  (bp) | Percentage  (%) | Length  (bp) | Percentage  (%) |
| **DNA** | 77,531,615 | 7.433187 | 15,595,537 | 1.495191 | 220,849,238 | 21.173475 | 254,286,466 | 24.379201 |
| **LINE** | 53,614,017 | 5.140136 | 36,423,847 | 3.492063 | 120,321,514 | 11.535582 | 149,506,486 | 14.333632 |
| **SINE** | 3,827,236 | 0.366929 | 0 | 0.000000 | 1,677,182 | 0.160796 | 5,412,750 | 0.518936 |
| **LTR** | 20,788,895 | 1.993093 | 11,968,456 | 1.147452 | 96,767,229 | 9.277363 | 105,756,307 | 10.139172 |
| **Other** | 14,851 | 0.001424 | 0 | 0.000000 | 108,562 | 0.010408 | 122,253 | 0.011721 |
| **Unkown** | 0 | 0.000000 | 0 | 0.000000 | 3,729,799 | 0.357587 | 3,729,799 | 0.357587 |
| **Total** | 149,259,513 | 14.309954 | 63,960,271 | 6.132062 | 409,385,596 | 39.249017 | 438,304,359 | 42.021545 |

**Supplementary Table 9** Statistics of function annotation

| Category Database | | Number | Percentage (%) |
| --- | --- | --- | --- |
| **Total** | | 23,611 |  |
| **Annotated** | InterPro | 21,888 | 92.70 |
|  | KEGG | 20,692 | 87.64 |
|  | Swissprot | 21,453 | 90.86 |
|  | TrEMBL | 23,477 | 99.43 |
| **Overall** |  | 23,492 | 99.50 |
| **Unannotated** | | 119 | 0.5 |

**Supplementary Table 10** Statistics of Non-coding RNAs in the genome

| **Type** | | **Copy** | **Average length (bp)** | **Total length (bp)** | **% of genome** |
| --- | --- | --- | --- | --- | --- |
| **miRNA** | | 247 | 82.4412955465587 | 20363 | 0.001952 |
| **tRNA** | | 2138 | 78.2212347988775 | 167237 | 0.016034 |
|  | rRNA | 538 | 124.421933085502 | 66939 | 0.006418 |
| **rRNA** | 18S | 36 | 294.694444444444 | 10609 | 0.001017 |
|  | 28S | 292 | 126.616438356164 | 36972 | 0.003545 |
|  | 5.8S | 4 | 129 | 516 | 0.000049 |
|  | 5S | 206 | 91.4660194174757 | 18842 | 0.001806 |
|  | snRNA | 298 | 131.137583892617 | 39079 | 0.003747 |
| **snRNA** | CD-box | 121 | 100.677685950413 | 12182 | 0.001168 |
|  | HACA-box | 59 | 153.322033898305 | 9046 | 0.000867 |
|  | splicing | 109 | 147.293577981651 | 16055 | 0.001539 |

**Supplementary Table 11** Statistics of gene families in 19 teleost fishes

| Species | Genes number | Genes in families | unassigned genes | Family number | Unique  family | Genes per family |
| --- | --- | --- | --- | --- | --- | --- |
| *A. limnaeus* | 20,204 | 19,353 | 851 | 16,382 | 33 | 1.18 |
| *A. testudineus* | 23,888 | 23,756 | 132 | 18,424 | 39 | 1.29 |
| *B. pectinirostris* | 20,854 | 20,590 | 264 | 17,042 | 23 | 1.21 |
| *C. argus* | 22,568 | 20,516 | 2052 | 16,953 | 66 | 1.21 |
| *C. variegatus* | 22,921 | 22,569 | 352 | 17,988 | 29 | 1.25 |
| *D. rerio* | 26,143 | 24,215 | 1928 | 16,793 | 333 | 1.44 |
| *F. heteroclitus* | 26,711 | 26,370 | 341 | 18,527 | 81 | 1.42 |
| *H. speculiger* | 23,611 | 22,083 | 1528 | 17,352 | 92 | 1.27 |
| *K. marmoratus* | 22,016 | 21,860 | 156 | 18,131 | 7 | 1.21 |
| *M. albus* | 20,859 | 20,595 | 264 | 17,381 | 29 | 1.18 |
| *M. zebra* | 25,861 | 25,698 | 163 | 18,705 | 38 | 1.37 |
| *N. furzeri* | 20,242 | 19,969 | 273 | 17,049 | 29 | 1.17 |
| *O. aureus* | 27,872 | 27,687 | 185 | 19,276 | 23 | 1.44 |
| *O. latipes* | 22,088 | 21,943 | 145 | 17,702 | 43 | 1.24 |
| *O. melastigma* | 22,826 | 22,518 | 308 | 17,833 | 40 | 1.26 |
| *P. magnuspinnatus* | 21,124 | 20,950 | 174 | 19,324 | 13 | 1.52 |
| *P. mexicana* | 25,040 | 24,586 | 454 | 17,391 | 59 | 1.2 |
| *X. maculatus* | 23,297 | 23,149 | 148 | 18,608 | 20 | 1.32 |
| *O. niloticus* | 29,754 | 29,448 | 306 | 16,382 | 66 | 1.18 |

**Supplementary Table 12** Statistics of pairwise alignment among mirrorwing flyingfish, medaka and zebrafish

|  | **Flyingfish vs medaka** | | **Flyingfish vs zebrafish** | |
| --- | --- | --- | --- | --- |
|  | Flyingfish | Japanese medaka | Flyingfish | Zebrafish |
| Total length | 1,043,046,751 | 734,057,086 | 1,043,046,751 | 1,679,203,469 |
| Matching length | 633,320,578 | 574,700,565 | 152,944,650 | 323,514,531 |
| Coverage (%) | 60.71 | 78.29 | 14.66 | 19.27 |

Note: The “net” output of LASTZ alignment was used to define large-scale syntenic regions, it allowed gaps and local small rearrangements.

**Supplementary Table 13** KEGG enrichment for genes of expanded gene families of the mirrorwing flyingfish.

| Pathway ID | Gene Numbers | KEGG Pathway name | P value |
| --- | --- | --- | --- |
| ko04080 | 51 | Neuroactive ligand-receptor interaction | 8.00E-08 |
| ko04727 | 50 | GABAergic synapse | 8.21E-24 |
| ko04621 | 49 | NOD-like receptor signaling pathway | 1.22E-19 |
| ko04721 | 48 | Synaptic vesicle cycle | 1.71E-26 |
| ko04514 | 39 | Cell adhesion molecules (CAMs) | 8.07E-11 |
| ko05164 | 36 | Influenza A | 7.88E-11 |
| ko03320 | 35 | PPAR signaling pathway | 1.02E-20 |
| ko04810 | 35 | Regulation of actin cytoskeleton | 2.85E-05 |
| ko04371 | 33 | Apelin signaling pathway | 1.08E-08 |
| ko04510 | 32 | Focal adhesion | 1.85E-03 |
| ko04923 | 32 | Regulation of lipolysis in adipocytes | 1.47E-15 |
| ko04070 | 30 | Phosphatidylinositol signaling system | 1.22E-09 |
| ko00310 | 29 | Lysine degradation | 5.15E-12 |
| ko04714 | 28 | Thermogenesis | 1.79E-04 |
| ko05100 | 28 | Bacterial invasion of epithelial cells | 5.48E-09 |
| ko05131 | 27 | Shigellosis | 2.30E-10 |
| ko04640 | 26 | Hematopoietic cell lineage | 4.93E-11 |
| ko04662 | 26 | B cell receptor signaling pathway | 4.76E-11 |
| ko04217 | 25 | Necroptosis | 1.28E-06 |
| ko04218 | 25 | Cellular senescence | 5.12E-04 |
| ko04625 | 24 | C-type lectin receptor signaling pathway | 8.31E-07 |
| ko05133 | 23 | Pertussis | 1.59E-08 |
| ko00534 | 21 | Glycosaminoglycan biosynthesis - heparan sulfate / heparin | 5.39E-14 |
| ko05162 | 19 | Measles | 7.54E-05 |
| ko04724 | 18 | Glutamatergic synapse | 3.49E-03 |
| ko01200 | 16 | Carbon metabolism | 2.68E-03 |
| ko05323 | 16 | Rheumatoid arthritis | 2.29E-05 |
| ko00140 | 15 | Steroid hormone biosynthesis | 2.59E-07 |
| ko00590 | 14 | Arachidonic acid metabolism | 1.11E-06 |
| ko04612 | 14 | Antigen processing and presentation | 2.59E-04 |
| ko04672 | 14 | Intestinal immune network for IgA production | 2.59E-07 |
| ko05150 | 14 | Staphylococcus aureus infection | 3.03E-06 |
| ko05320 | 14 | Autoimmune thyroid disease | 3.89E-06 |
| ko00020 | 13 | Citrate cycle (TCA cycle) | 2.59E-07 |
| ko00620 | 13 | Pyruvate metabolism | 5.63E-06 |
| ko05211 | 13 | Renal cell carcinoma | 1.64E-03 |
| ko05322 | 13 | Systemic lupus erythematosus | 1.60E-03 |
| ko05310 | 12 | Asthma | 1.04E-07 |
| ko05330 | 12 | Allograft rejection | 3.24E-05 |
| ko00592 | 10 | alpha-Linolenic acid metabolism | 3.10E-07 |
| ko00830 | 10 | Retinol metabolism | 7.04E-04 |
| ko04940 | 10 | Type I diabetes mellitus | 1.64E-03 |
| ko05144 | 10 | Malaria | 2.34E-03 |
| ko05321 | 10 | Inflammatory bowel disease (IBD) | 2.61E-03 |
| ko05332 | 10 | Graft-versus-host disease | 2.70E-04 |
| ko00130 | 9 | Ubiquinone and other terpenoid-quinone biosynthesis | 9.34E-08 |
| ko01040 | 9 | Biosynthesis of unsaturated fatty acids | 3.16E-04 |
| ko00040 | 7 | Pentose and glucuronate interconversions | 1.72E-03 |
| ko00053 | 7 | Ascorbate and aldarate metabolism | 3.64E-04 |
| ko00860 | 7 | Porphyrin and chlorophyll metabolism | 3.59E-03 |
| ko00980 | 7 | Metabolism of xenobiotics by cytochrome P450 | 2.34E-03 |
| ko00982 | 7 | Drug metabolism - cytochrome P450 | 2.34E-03 |
| ko04136 | 7 | Autophagy - other | 6.62E-03 |
| ko04215 | 7 | Apoptosis - multiple species | 6.62E-03 |
| ko05204 | 7 | Chemical carcinogenesis | 6.62E-03 |
| ko00591 | 6 | Linoleic acid metabolism | 2.34E-03 |

**Supplementary Table 14** Estimated maximal absorption spectrum (λ_max_) of LWS

| species | Opsin type | Tuning Site | | | | | Estimated λ_max_ |
| --- | --- | --- | --- | --- | --- | --- | --- |
|  |  | 180 | 197 | 277 | 285 | 308 |  |
| *Ancestor* | LWS | S | H | Y | T | A | 560 |
| *H. sapiens* | LWS | A | H | Y | T | A | 553 |
|  | MWS | A | H | F | A | A | 531 |
| *A. testudineus* | LWS1 | S | H | Y | T | A | 560 |
|  | LWS2 | S | H | Y | T | A | 560 |
| *B. pectinirostris* | LWS1 | A | H | Y | T | A | 553 |
|  | LWS2 | A | H | F | A | A | 531 |
| *P. magnuspinnatus* | LWS1 | S | H | Y | T | A | 560 |
|  | LWS2 | A | H | F | T | A | 546 |
| *C. argus* | LWS1 | S | H | Y | T | A | 560 |
|  | LWS2 | S | H | Y | T | A | 560 |
| *H. speculiger* | LWS1 | S | H | Y | T | A | 560 |
|  | LWS2 | S | H | Y | T | A | 560 |
| *K. marmoratus* | LWS1 | S | H | Y | T | A | 560 |
|  | LWS2 | S | H | Y | T | A | 560 |
| *O. melastigma* | LWS1 | A | H | Y | T | A | 553 |
|  | LWS2 | S | H | Y | T | A | 560 |
| *O. latipes* | LWS1 | S | H | Y | T | A | 560 |
|  | LWS2 | S | H | Y | T | A | 560 |
| *O. aureus* | LWS | S | H | Y | T | A | 560 |
| *O. niloticus* | LWS | S | H | Y | T | A | 560 |
| *M. zebra* | LWS | A | H | Y | T | A | 553 |
| *D. rerio* | LWS1 | A | H | Y | T | A | 553 |
|  | LWS2 | A | H | F | T | A | 546 |

**Supplementary Table 15** Copy numbers of intact OR genes in each group of fishes and mammals

|  | Species | Water | | | | Air | | Air/  Water | Non-OR | Total |
| --- | --- | --- | --- | --- | --- | --- | --- | --- | --- | --- |
|  |  | Delta | Epsilon | Zeta | Eta | Alpha | Gamma | Beta | Kappa |  |
| Aquatic | *A. testudineus* | 100 | 9 | 10 | 19 | 0 | 0 | 7 | 0 | 145 |
|  | *B. pectinirostris* | 43 | 3 | 4 | 1 | 0 | 0 | 1 | 1 | 53 |
|  | *P. magnuspinnatus* | 37 | 9 | 11 | 2 | 0 | 0 | 1 | 1 | 61 |
|  | *C. argus* | 116 | 5 | 10 | 3 | 0 | 0 | 7 | 0 | 141 |
|  | *K. marmoratus* | 32 | 5 | 3 | 7 | 0 | 0 | 3 | 0 | 50 |
|  | *H. speculiger* | 18 | 7 | 5 | 19 | 0 | 0 | 1 | 0 | 50 |
|  | *D. rerio* | 30 | 19 | 11 | 24 | 0 | 1 | 7 | 1 | 93 |
|  | *O. aureus* | 102 | 9 | 8 | 24 | 0 | 0 | 6 | 0 | 149 |
|  | *O. latipes* | 24 | 3 | 5 | 7 | 0 | 0 | 3 | 1 | 43 |
| Terrestrial | *B. taurus* | 0 | 0 | 0 | 0 | 140 | 828 | 2 | 1 | 971 |
|  | *H. sapiens* | 0 | 0 | 0 | 0 | 58 | 329 | 0 | 0 | 387 |
